# Thermodynamic Biomarkers of Neuroinflammation: Nanothermometry, Energy–Stress Dynamics, and Predictive Entropy in Glial–Vascular Networks

**DOI:** 10.3390/ijms262211022

**Published:** 2025-11-14

**Authors:** Valentin Titus Grigorean, Adrian Vasile Dumitru, Catalina-Ioana Tataru, Matei Serban, Alexandru Vlad Ciurea, Octavian Munteanu, Mugurel Petrinel Radoi, Razvan-Adrian Covache-Busuioc, Ariana-Stefana Cosac, George Pariza

**Affiliations:** 1 Puls Med Association, 051885 Bucharest, Romania; 2 Faculty of General Medicine, “Carol Davila” University of Medicine and Pharmacy, 050474 Bucharest, Romania; 3 Department of Pathology, Faculty of Medicine, “Carol Davila” University of Medicine and Pharmacy, 030167 Bucharest, Romania; 4 Clinical Department of Ophthalmology, “Carol Davila” University of Medicine and Pharmacy, 020021 Bucharest, Romania; 5 Department of Ophthalmology, Clinical Hospital for Ophthalmological Emergencies, 010464 Bucharest, Romania; 6 Department of Neurosurgery, “Carol Davila” University of Medicine and Pharmacy, 050474 Bucharest, Romania; 7 Department of Vascular Neurosurgery, National Institute of Neurology and Neurovascular Diseases, 077160 Bucharest, Romania; 8 Medical Section, Romanian Academy, 010071 Bucharest, Romania; 9 Neurosurgery Department, Sanador Clinical Hospital, 010991 Bucharest, Romania; 10 5th Department of General Surgery, Emergency Hospital Bucharest, 050098 Bucharest, Romania

**Keywords:** neuroinflammation, thermodynamic biomarkers, energetic coherence, entropy flux, redox oscillations, nanothermodynamic therapy, mitochondrial coupling, glial–vascular synchronization, predictive neuroenergetics, quantum thermometry

## Abstract

Homeostasis, which supports and maintains brain function, results from the continuous regulation of thermodynamics within tissue: the balance of heat production, redox oscillations, and vascular convection regulates coherent energy flow within the organ. Neuroinflammation disturbs this balance, creating measurable entropy gradients that precede structural damage to its tissue components. This paper proposes that a thermodynamic unity can be devised that incorporates nanoscale physics, energetic neurophysiology, and systems neuroscience, and can be used to understand and treat neuroinflammatory processes. Using multifactorial modalities such as quantum thermometry, nanoscale calorimetry, and redox oscillometry we define how local entropy production (s_t_), relaxation time (τ^R^), and coherence lengths (λc) allow quantification of the progressive loss of energetic symmetry within neural tissues. It is these variables that provide the basis for the etiology of thermodynamic biomarkers which on a molecular-redox-to-network scale characterize the transitions governing the onset of the neuroinflammatory process as well as the recovery potential of the organism. The entropic probing of systems (PEP) further allows the translation of these parameters into dynamic patient-specific trajectories that model the behavior of individuals by predicting recurrent bouts of instability through the application of machine learning algorithms to the vectors of entropy flux. The parallel development of the nanothermodynamic intervention, which includes thermoplasmonic heat rebalancing, catalytic redox nanoreacting systems, and adaptive field-oscillation synchronicity, shows by example how the corrections that can be applied to the entropy balance of the cell and system as a whole offer a feasible form of restoration of energy coherence. Such closed loop therapy would not function by the suppression of inflammatory signaling, but rather by the re-establishment of reversible energy relations between mitochondrial, glial, and vascular territories. The combination of these factors allows for correction of neuroinflammation, which can now be viewed from a fresh perspective as a dynamic phase disorder that is diagnosable, predictable, and curable through the physics of coherence rather than the molecular suppression of inflammatory signaling. The significance of this set of ideas is considerable as it introduces a feasible and verifiable structure to what must ultimately become the basis of a new branch of science: predictive energetic medicine. It is anticipated that entropy, as a measurable and modifiable variable in therapeutic “inscription”, will be found to be one of the most significant parameters determining the neurorestoration potential in future medical science.

## 1. From Inflammation to Entropy: The Thermodynamic Collapse of Neural Predictability

### 1.1. Experimental Rationale

While neuroinflammation is typically considered due to cytokine cascades, reactive oxygen species, and mitochondrial dysfunction, it represents the end result of a larger problem related to energy: the lack of predictability in the energy used by mitochondria, transported throughout the glial–vascular system and dissipated [1]. Under normal conditions, the brain maintains a rhythmic energy homeostasis in contrast to being stationary. Therefore, oxidative phosphorylation by mitochondria, glycolysis by astrocytes, ion fluxes, and vasomotor oscillations functionally synchronize to create reversible energy flow where the entropy produced by neural computations is returned via both mitochondrial and vascular feedback mechanisms [2,3,4]. As a result, the synchronized interaction between oxygen flux, heat produced, and synaptic computations maintains a dynamic equilibrium state. However, inflammation occurs once the typical time frames for oxidation, perfusion, and electrical signal transduction lose synchronization [5].

Activated microglia and astrocytes generate energetic “noise” by transitioning from oxidative phosphorylation to glycolysis during activation. As a result, they increase the amount of entropy generated and decrease the reversibility of redox reactions. Additionally, proton leakage through damaged inner mitochondrial membranes generates measurable millikelvin fluctuations in local temperatures measured by nanoscale thermometry [6]. Localized thermal microbursts then travel through the cytosol and extracellular matrix disrupting ionic gradient and synchrony among cell populations [7]. Typically, Δψm oscillate in synchrony among all neuronal and glial cells, but once oxidative stress is introduced phase slips occur and the spectral distribution of Δψm becomes broader, causing bioenergetic oscillators to operate in an asynchronous manner with reduced reversibility [8]. Asynchronous bioenergetic oscillators reduce the functional coherence between the neural tissue and its vascular supply, reducing neurovascular coupling and delaying hemodynamic responses and also disrupting astrocyte calcium signaling resulting in reduced cerebral perfusion and utilization of energy [9].

### 1.2. Validation of Models

Both neuroimaging and electrophysiology data provide evidence to support this thermodynamic model. Data obtained using fMRI and optical recording techniques indicate an increase in oxygen extraction fraction (OEF) in excess of glucose metabolism, indicating a separation in oxidative efficiency from substrate consumption [10]. Electrophysiological spectra show reduced power density in cortical rhythms and reduced alpha and beta oscillations, demonstrating decreased coherent energy exchange and increased entropy. Avalanche dynamics have been shown to shift away from critical distributions to supercritical distributions, indicating an increase in the cost of energy expended by neurons to produce the same amount of information and thus a hallmark of dissipative pathology. Molecular biological markers that were traditionally used to diagnose neuroinflammation can be viewed as symptoms of an underlying thermodynamic imbalance. Increased lactate levels represent irreversibly consumed glycolytic energy; elevated cytokines represent futile energy cycles and increased Translocator Protein (TSPO) represents proton leakage through uncoupled electron transport chains. All three types of change demonstrate a single physical process: the generation of entropy at a rate greater than the glial and vascular system’s ability to absorb that entropy [10,11].

To further validate the results from this study, an overall literature search across Web of Science, PubMed, and Scopus was conducted for articles published between January 2000 and June 2025. The literature search consisted of all possible combinations of the following search terms; “neuroinflammation,” “thermodynamics,” “entropy,” “bioenergetics,” “nanothermometry,” “quantum sensors,” and “energy metabolism.” Articles had to provide either quantitative or model-based information regarding energetic imbalance, entropy generation, or coherence dynamics in neural tissues. Articles based solely on molecular or immunologic data and lacking an energetic context were excluded. Using the methodology described above, it was ensured that the theoretical underpinnings of this study are based upon reproducible empirical data and that all referenced studies provide measurable, thermodynamic correlates of neuroinflammatory processes.

### 1.3. Implications for Clinical Practice

A thermodynamic view of neuroinflammation presents neuroinflammation as an energy imbalance rather than simply a chemical imbalance—a state in which the rates of chemical reaction exceed the rate of regulatory feedback in order to restore balance. New nanoscale measurement tools allow for the measurement of these dynamic processes. For example, nanoscale thermometers based on up-conversion nanoparticles or nitrogen-vacancy centers in diamonds can measure heat fluxes generated by sub-cellular processes with a temporal resolution of milliseconds; photoacoustic nanocalorimetry can quantify microjoule-scale energy pulses from activated glia; and two-photon redox lifetime imaging can measure the phase relationship between NADH and FAD [12,13]. This will allow neuroinflammation to be directly measured as a thermodynamic process and thus the use of entropy as a biomarker [14].

New quantitative measures such as entropy production rate (σ), redox phase locking value (Φ_e_), and energy recovery half-life (τ^R^) will now describe how quickly a neural population can recover predictive energy patterns following some form of energetic perturbation. These new measures will describe the resilience of the neural population at many scales from molecular biochemistry to systems physiology [15].

The goal of this new framework is to bring together recent developments in nanothermometry, magnetic/photothermal perturbations, and dynamic stress testing into a common language for diagnosis. The measurement of predictability of energy rather than the concentration of metabolites should express neuroinflammation as a reversible deviation in energy coherence—measurable in terms of entropy, temperature, and time.

Here, various physical measures take on unique biological meanings. The entropy generation rate (σ) indicates how much metabolic reactions produce an irreversible type of heat; it is a measure of the degree of energetic disorder produced in neural tissue. The redox phase-locking value (Φ_e_) measures the synchronization of oxidative and reductive phases of mitochondrial metabolism among different populations of mitochondria, thus representing the degree to which cellular populations share an “energetic tempo”. The energy recovery half-time (τ^R^), or “time-to-recovery”, represents the average amount of time required for a neural population to return to thermal equilibrium after some form of perturbation, and provides a simple, practically useful measure of the resilience of that population’s energetic function. In combination with one another, σ, Φ_e_, and τ^R^ represent a set of physical terms and concepts with which inflammation can be characterized as the result of not only the relative abundance of inflammatory molecules, but also the organization, rhythms, and recoverability of energy itself.

## 2. Thermodynamic Architecture of Neuroinflammatory Energy Flow

### 2.1. Redox Thermodynamics and the Microstructural Origins of Entropy Production

Neuroinflammation is the process of converting the brain’s energetic circuits from a coherent thermodynamic system to a dissipative system in which redox and vascular systems no longer operate in a synchronized manner [16,17]. Mitochondria are the primary site for entropy regulation in the brain, maintaining reversible coupling between chemical potential (ΔG) and ATP generation through coherent electron transport across complexes I–IV [18]. Mitochondrial NADH/NAD^+^ and FAD/FADH_2_ potential fluctuation remain in-phase with oxygen consumption, ensuring reversible proton–electron coupling. Activation of inflammatory pathways disrupts this phase coherence. Reactive oxygen and nitrogen species inhibit electron transport and increase the fluorescence lifetime of NADH, disrupting the resonance between redox centers [19,20]. “Redox flicker” events occur on the picosecond–nanosecond time scales and reduce tunneling efficiency through Fe-S clusters, resulting in localized heat burst and increased mitochondrial entropy production (σmit) above its physiological minimum. Oxidative damage to cardiolipin alters the membrane curvature and generates viscous domains with randomly packed lipids that produce millikelvin “thermal grain” following non-Gaussian Levy statistics of turbulent diffusion [21,22,23,24,25]. Thus, mitochondria become non-ergodic ensembles. DRP1-mediated hyperfission increases the entropy output by fragmenting the reticulum, increases the surface-to-volume ratio, and increases proton leaks; calorimetry has shown transient doubling of local heat fluxes due to each fission event [26,27].

Electron coherence lifetimes decrease from hundreds of femtoseconds to tens of femtoseconds, demonstrating the inefficiency in energy conversion and irreversible sub-cellular heat flow [28,29].

### 2.2. Validation of Models: Gliovascular Coupling as an Entropic Regulator

**Figure 1:**
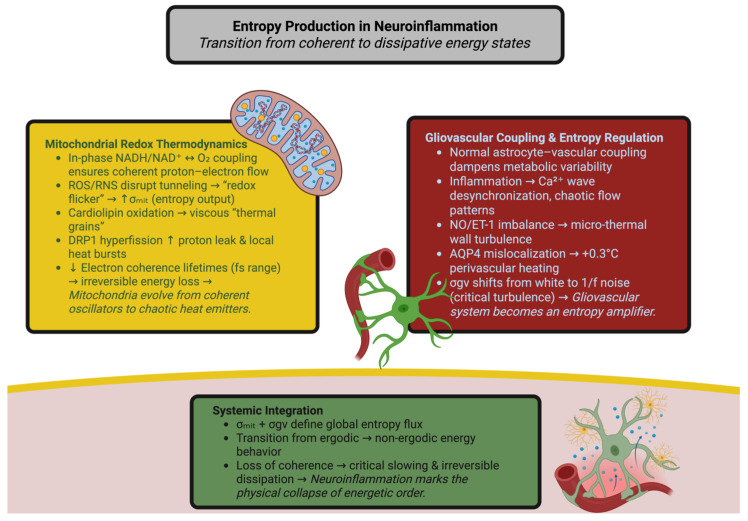
Entropy production in neuroinflammation: transition from coherent to dissipative energy states. The illustration depicts how inflammation of the nervous system transforms the brain’s redox–vascular network from a coherent thermodynamic system to a dissipative one. On the left, the disruption of mitochondria via ROS, cardiolipin oxidation, and DRP1 hyperfusion leads to increased entropy production of mitochondria (σmit). On the right, gliovascular desynchronization as well as mislocalization of AQP4 increase local turbulence (σgv). At the bottom, the combination of both processes contributes to global entropy flow and loss of coherence, with eventual physical collapse of energetic organization.

Astrocytes, microglia, and endothelial cells act as a dynamic entropic controller that links metabolic activity and perfusion. In normal conditions, Ca^2+^-dependent gliotransmission and pericyte contraction help to dampen metabolic variability; however, inflammation turns this controller into a random amplifier. Astrocytic Ca^2+^ waves lose spatial coherence and propagate at velocities reduced from hundreds of micrometers to tens of micrometers, and their correlation function changes from a power law to exponential decay—indicating the breaking down of self-similarities [30,31,32]. Perfusion signals become chaotic; cytokine-induced nitric oxide/endothelin co-phasing breaks down vascular resonance and creates micro-thermal wall fluctuations [33]. Astrocytic swelling and aquaporin-4 mislocalization increase the residence time of heat around the vessels by over 60% and therefore raise the temperature by 0.2–0.3 °C during chronic inflammation [34]. Impaired removal and altered solubility of gases enhance feedback instability [35]. Therefore, gliovascular coupling transforms from a damped to a resonant behavior, its dissipation spectrum (σgv) changing from white to 1/f noise with long-range correlations characteristic of critical turbulence [36,37,38]. Such long memory characteristics of behavioral manifestations of the transitional state of stability to irreversible decay are thermodynamically comprehensible in entropy–power metrics’ methodology of fluctuations of temperature and flow [39]. Figure 1 illustrates how the brain’s energy system transitions from being coherent to being dissipative due to a neuroinflammatory response. The entropic effects of mitochondrial redox instability and gliovascular desynchronization serve as coupled sources of entropy in the brain that disrupt both proton–electron balance and vascular thermoregulation. Together these two provide the conditions for a systemic transition from reversible energy flow to irreversible heat loss—a thermodynamic hallmark of neuroinflammatory degradation.

### 2.3. Implications for Clinical Practice: Multiscale Entropy Flux and Diagnostic Application

The transmission of entropy in neuroinflammation follows hierarchical coupling, ranging from quantum decoherence to macroscopic dissipation [40].

Localized heat pulses generated within the mitochondrial membranes as a result of the piezoelectric actin–ion coupling and transmitting them to the cell membrane are detectable by atomic force microscopy; these pulses have increased amplitude in inflamed astrocytes [41]. In normal conditions, connexin-43 networks distribute the fluctuations and create an efficient heat conductance; however, inflammation breaks up these networks and generates isolated “thermal islands” visible in ratiometric nanothermometry [42,43].

On the mesoscale level, irregular heat conductance increases the phase dispersion of theta–beta coupling and disconnects metabolic oscillators from electrical oscillators, decreasing the predictive entropy of the system—the system’s capability to predict its next energetic state [44,45].

On the macroscale level, the entropy current density (Js) becomes a single unified disease metric. The use of MRI thermometry has demonstrated the existence of scale-invariant Js fluctuations and decreased fractal dimension (Dt) with increasing disease severity, approaching Dt = 2.0 in advanced inflammation. This loss of fractal dimension corresponds to the cortical spectral “whitening”, in which the 1/f structure of the cortical spectra collapses to the thermal noise [46,47]. To clarify these multiscale observations, Table 1 is presented to clarify a thermodynamic hierarchy whereby ordered flow processes are transfigured in terms of dissipative chaotic processes incurred in neuroinflammation.

Entropy conduction at present is not merely theoretical, in that it can be measured and evaluated. The quantities of temperature deviation, of heterogeneity in redox lifetimes, of loss of phase-locking in NADH cycles, and of deviation of viscosity in relation to perivascular space all indicate entropy conduction at different scales. Together they make up a basis for calculating a cross-scale Entropy Transfer Function (ETF), which in general will be based on nascent thermometry, mesoscopic photoacoustics, and microscopic hemodynamics of energy transfer. In reviewing the ETF, it emerges that during the course of neuroinflammation the time constants for energy coherence are diminished at all levels, which in turn is true from femtoseconds in redox processes to seconds in vascular oscillations, and indicates in this a universal loss of temporal correlation in the energy conduction process [61].

This whole mentalistic process of the progress of thermodynamic degradation forms the physical, measurable answer to the problem of the progress of neuroinflammation. Each one of these deviations entails a sum of minor order which quite recedes into the space–time of the process as a whole and degenerates correspondingly into irreversibility; that is to say, a state of affairs in which the transformation of the energy no longer predicts its own future results [62]. By means of the reduction in the magnitude to be measured it is possible to transmute the state called inflammation from a qualitative condition to something that can be physically represented by the laws of measurable entropy production, diffusion, and coherence. Considered in this light, the inflamed brain is a nonequilibrium thermodynamic storage unit whose pathology may be described not by the operations of molecules, but the physical properties of energy itself [63].

This interrelationship shows that both thermodynamic unity and energetic coherence are different expressions for one physical reality. Thermodynamic unity describes the continuous exchange of all energy transactions occurring throughout the neural network (electron transfers from mitochondrial energy production to oscillating blood perfusion through vascular networks) and forms a single, open system of dynamic equilibrium. Energetic coherence describes the time and space expression of this unity and is the condition in which local changes occur in synchronization with the global energy flux. If the unity of the brain remains intact, then reversible transitions will continue to exist between metabolic and electrical activities; however, if this unity is lost, then the coherence of the brain will collapse and lead to an increase in entropy and functional decline. Therefore, neuroinflammation can be quantitatively measured as a fragmentation of thermodynamic unity and therefore loss of coherence that converts a coordinated energy exchange into random or disordered processes at the molecular, cellular, and systemic levels.

Translating this theoretical model into an experimental context will require instrumentation that can measure energy transformations occurring in real time inside the body. Nanothermodynamics is the developing field that offers this translation by turning abstract measures of entropy generation (and loss) into measurable physical phenomena. Optical, magnetic, and calorimetric instrumentation at the nanoscale level enables tracking of the disintegration of energetic order as it occurs throughout all levels of neuroinflammatory response (from molecular fluctuation through vascular dynamic). These instrumentation capabilities convert what has been abstractly conceptualized as entropy into a measurable biological quantity, thus providing the empirical underpinnings for the subsequent sections.

## 3. Nanothermometric Framework for Mapping Thermodynamic Biomarkers

The ability to perceive energy directly—to see its transmission, its plasticity, and ultimately its loss—represents an enormous leap forward in neuroinflammatory research. Traditional imaging methods show metabolic indicators: glucose uptake, oxygen utilization, receptor binding, and cytokine distribution. However, nanothermometry has the capability to actually measure the energy itself: the conversion of free energy into randomness, and the physical manifestation of inflammation as entropy [64].

Healthy brains have a rhythmic dance of energy flux—oxidation, diffusion, perfusion—which is reversible and coherent. Inflammation disrupts this dance and creates a cycle of redox phase loss, increased viscosity, and the presence of disordered thermal vibrations. The purpose of nanothermometry is to quantify and describe the microphysical manifestations of inflammation, including those related to entropy, coherence, and resilience [65,66,67].

There are many emerging techniques currently being developed, one of which is up-conversion nanothermometry, which offers optical access to sub-cellular energy behaviors. Lanthanide doped up-conversion nanoparticles (UCNPs)—typically NaYF4:Yb3+, Er3+, Ho3+—convert infrared light into visible light by absorbing multiple photons, where the ratio of thermally coupled states is governed by a Boltzmann distribution, producing <0.02 °C sensitivity tens of mm deep in neural tissue [68]. These UCNPs serve as both thermometers and thermodynamic translators, when encapsulated in mesoporous shells containing redox sensitive materials, such as anthraquinones or viologen derivatives; they create thermo–redox phase diagrams that represent the relationship between heat dissipation and oxidative stress [69].

In healthy tissue, the temperature and redox potential oscillates within tight bands; however, during inflammation, this coherence disappears and a hysteresis loop—a nonequilibrium indicator of the transition from reversible metabolism to dissipative metabolism—forms. Gold or silver plasmonic nanoshells added to the surface of UCNP cores allow for a perturbation, creating microjoules of localized heat while measuring the recovery kinetics of the up-converted emission. The energy recovery half-time (τ^R^) represents the amount of time required for a region of tissue to regain thermal equilibrium post-stress. In the healthy cortex, τ^R^ = 0.7 ± 0.2 s, whereas in inflamed areas it is greater than 4 s—indicative of diminished thermal conductivity and redox flexibility [70,71]. Advanced systems of co-doping (Nd^3+^, Dy^3+^) can reference themselves against the effects of scattering or photobleaching, and provide millikelvin accuracy for long durations of imaging. These constructs allow for the direct measurement of entropy production in clusters of microglia and in perivascular microdomains where energy gradients are best reflected [72,73].

Additional information about inflammation may be gained from quantum diamond thermometry. Nitrogen-vacancy (NV) centers in diamond nanocrystals function as spin-based sensors, whose resonance frequency is linearly shifted with respect to temperature (~74 kHz/K). Using optical detection of magnetic resonance (ODMR), spatial resolutions less than 100 nm and thermal sensitivities less than 50 mK are achievable [74]. In addition to temperature, NV sensors measure fluctuations in local magnetic and electric fields and relate redox reactions to thermodynamic noise at a quantum level. Reactive oxygen bursts cause broadening of the ODMR line-width (Δν), and lengthen the spin relaxation time; the rate of this broadening (dΔν/dt) directly correlates with the rate of entropy production (σ). In regions rich in microglia, Δν values of 0.3–0.5 MHz indicate an increase in σ of 30–50% compared to adjacent normal tissue [75]. Therefore, NV thermometry shows magnetothermal coupling—the synchronized interaction between heat and magnetic fluctuations—and indicates the loss of reversibility of local redox equilibria. In inflamed areas, temperature and magnetic noise are out of phase by approximately 20 ms, similar to mitochondrial proton leak kinetics and representing the local loss of reversible energy absorption [76,77].

When combined with confocal or multiphoton microscopy, NV recordings can generate four dimensional tensors representing the energetic properties of tissue, including thermal, magnetic, and redox properties. Longitudinal studies in animal models have shown that inflammation spreads not only through chemical cascade pathways but also through field-like interactions throughout the tissue [78].

A further aspect of thermodynamic behavior—mechanical dissipation—is measured by AIE probes functioning as nanoscale viscometers. Viscosity in healthy astrocytes is maintained within the range of 2–3 cP; however, inflammation increases it by more than 40% through oxidative crosslinking and cytoskeleton condensation [79,80]. AIE luminogens respond to an increase in fluorescence as molecular rotation decreases, and thereby convert cells into microscopic reporters of mechanical stiffness. Hybrid europium-based AIE nanoparticles produce red Eu3+ emission lines proportionally to temperature and blue emission lines proportionally to viscosity, allowing for the definition of the thermoviscous coefficient (Θv)—the fraction of energy lost as mechanical deformation [81].

These thermodynamic signatures may be characterized with high precision as well as temporal resolution to locally record and perturb, as local entropy generation (σ) is determined from time-resolved transient heat flux measurements made possible via UCNP or NV-diamond sensor measurements and related to conductive flows (Jq) as defined by the equation σ = Jq·∇(1/T) [75]. Energy recovery half times (τ^R^) and thermal conductivity (κ) are also determined by fitting an exponential curve to the temperature changes resulting from a nanosecond scale photothermal or magnetothermal pulse that determines the elasticity of the local energy flow. Finally, coherence length (ξc) and phase locking values are determined by performing a cross correlation between redox or thermal oscillations occurring across adjacent regions to determine the distance at which energetic fluctuations remain correlated. Collectively these values provide a methodical approach to experimentally map the reversibility, coupling, and entropy associated with the flow of energy within the nervous system in both physiologic and inflamed states [82].

Photoacoustic nanocalorimetry complements these measurements by detecting ultrasound echoes produced by picosecond thermal expansion and provides a measurement of the thermal elastic modulus (Et)—a measurement of the density of mitochondria and membrane fluidity. Areas of high Et are markers of entropic attractors—areas where residual heat and biochemical noise accumulate, and form the structural basis for chronic inflammation [83,84].

Calibration among these methods allows for quantitative integrity. Corrections for the emission of UCNPs are made using ratiometric AIE or rhodamine standards; calibration of NV sensors uses dual-frequency microwave modulation to reduce the effects of strain artifacts. Integration of nanoscale τ^R^, Θv, and Et with macroscopic metrics such as BOLD-fMRI perfusion, ^31^P-MRS ATP turnover, and PET-TSPO microglial activity demonstrates significant covariation between microthermal noise and systemic metabolism [85]. Three major thermodynamic axes are identified through principal component analysis: (i) τ^R^ and Et—reflect the capacity of entropic recovery; (ii) Θv and Ds—describe the relationship between viscosity and diffusion; and (iii) phase locking metrics—combine redox, thermal, and magnetic coherence [86,87]. These axes collectively define the Thermodynamic State Space (TSS)—a multidimensional space of all possible tissue energy behaviors ranging from reversible adaptation to irreversible degeneration. From this, the Composite Thermodynamic Map (CTM) is defined—a spatiotemporal atlas that combines optical, quantum, and mechanical data to provide a coherent representation of energy flow topology. CTM imaging demonstrates that areas with prolonged τ^R^ and increased Θv often occur before evident structural damage by months—and converts inflammation from an abstract biochemical concept to a physically measurable process [88].

**Figure 2:**
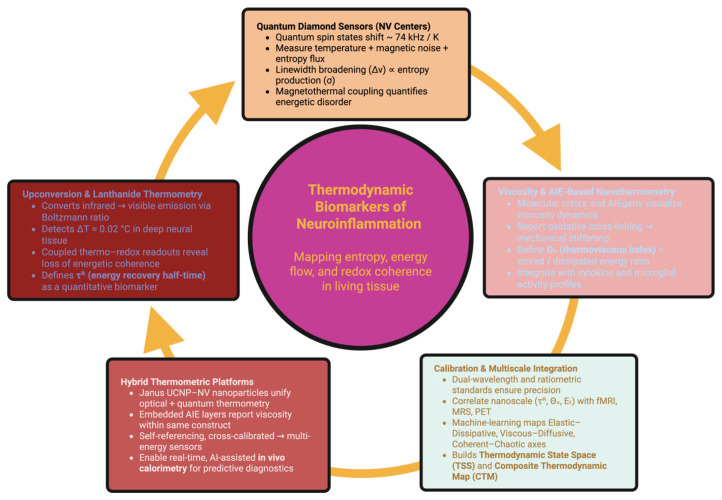
Nanothermometric framework for mapping thermodynamic biomarkers. This schematic integrates optical, quantum, and mechanical nanosensing modalities for detecting energetic disorder in neuroinflammation. The central node represents thermodynamic biomarkers—quantifiable signatures of entropy, energy flow, and redox coherence in living tissue.

Figure 2 is a representation of the coupling of nanothermal measurement and its integration into a single unifying physical framework, and how the energetic behaviors can be seen as a homogenous continuous landscape defining the characteristics ranging from stability of high energy coherent behavior to disorder seen in the presence of neuroinflammation.

These technologies will soon allow for the development of multifunctional “Janus” nanoparticles—each having both UCNP and NV-diamond and AIE elements within the same hybrid sensor. This combination will allow for simultaneous optical and quantum readout of temperature, redox state, viscosity, and magnetic noise—and allow for real-time, self-referential thermodynamic characterization [89]. Embedded in flexible hydrogels or neural scaffolds, these probes will allow for continuous in vivo calorimetry and the use of wireless optical or magnetic signal transmission. Continuous measurement of the flux of entropy will allow for the implementation of closed-loop thermodynamic diagnostic systems (OT/TD systems)—where AI assisted analytics will identify early changes in the baseline energetic coherence of the tissue and predict the onset of inflammatory responses [90]. Within this new framework, nanothermometry moves from being a method of measurement to a physical language to describe how the brain retains or loses energetic equilibrium. Through the use of photons, spins, and acoustic pulses, we tell one story: inflammation is the point at which energy stops being rhythmic—and the restoration of this rhythm is the physical process of healing [91].

Nanometer-scale temperature fluctuations associated with synaptic bursting have been demonstrated by using nanothermometric techniques in isolated, optically transparent ex vivo brain slices [92]. Temperature fluctuations of less than 1 degree Celsius were observed in discrete clusters of neurons; these fluctuations occurred when there was synchronized burst firing of action potentials in specific neuronal populations and were inversely related to the mitochondrial redox potential of those cells [93,94]. The data suggest that the metabolic processes that generate heat are not uniformly distributed throughout the cell or tissue; rather they occur in organized patterns of oscillating clusters of oxidative metabolism and ion flux [95]. This has been confirmed by use of quantum diamonds containing nitrogen-vacancy (NV) centers to map thermal and magnetic noise at the sub-cellular level in living preparations. When NV-doped nanodiamonds were used to measure the magnetic and thermal noise generated by inflammatory activated cells, the data showed broadening of the spectral lines and shifting of their time of occurrence; this indicated an increased rate of entropy generation and a loss of redox phase coherence across adjacent glial and vascular microdomains [96]. Hybrid methods combining up-conversion thermometry, magnetic resonance, and redox lifetime imaging have now allowed measurement of the energetic transitions in the brain in 4D space–time, showing that localized heat loss due to cellular metabolism can result in systemic effects such as changes in vascular blood flow and oxygen delivery. Thus, it appears that the loss of energetic predictability during inflammation is an empirical phenomenon that is measurable as a function of both location and time in the living brain; this loss of predictability is equivalent to an increase in entropy and can thus be measured as an empirical spatial–temporal property of the living brain [97].

Having confirmed that the degradation in energetic predictability due to inflammation can be measured, the subsequent goal is to ascertain the degree to which the neural system is able to withstand energetic disruptions. Rather than simply observing the production of disorder (entropy), it becomes imperative to investigate how the brain reacts to induced energetic disruptions—to assess both disorder and recoverability. Perturbational Energy–Stress Phenotyping has developed as an answer to the demand for assessing the capacity of the nervous system to respond to induced energetic disruptions—and therefore assesses the degrees of resilience, reversibility, and coherence by measuring the energetic response to experimental micro-disruptions. As such, it converts thermodynamics to a practical assessment of the brain’s capacity to restore energetic order after experiencing stress.

## 4. Perturbational Energy–Stress Phenotyping: Quantifying Neuroenergetic Resilience

Neuroinflammation signifies an energetic and functional disruption beyond mere overactive immune responses; it is a gradual disintegration of the brain’s ability to recover from energetic disruptions. Essentially, neuroinflammation is a failure in dynamic reversibility—the inability of the neural tissue to absorb entropic stress and to create coherence throughout all of the redox, electrical, and vascular domains. Perturbational Energy–Stress Phenotyping (PESP) has provided the first quantitative approach to addressing this issue by providing controlled micro-scale energy pulses and examining recovery kinetics. Every perturbation—magnetic, photothermal, or electric—acts as a thermodynamic question posed to the tissue, and the rapidness and symmetry of the tissue’s response will be indicative of the healthiness of the underlying neuroenergetic network [98,99,100].

Magnetothermal and photothermal perturbations probe the elasticity of energy flow at the microscopic scale. When superparamagnetic iron oxide nanoparticles (SPIONs) are embedded in astrocytic networks and subjected to oscillating magnetic fields, SPIONs generate nanoscale thermal bursts of 1–5 µJ (Néel-type thermal events) without exceeding physiological temperatures [101,102]. Simultaneously, the transient changes in localized temperature, redox potential, and osmolarity are recorded by concurrent nitrogen-vacancy diamond sensors that enable real-time thermodynamic mapping [103]. Healthy cortex will display single exponential relaxation curves post perturbation, demonstrating that the thermal and biochemical processes recover rapidly and in tandem. In contrast, inflamed tissues will display multiexponential relaxation curves: heat will dissipate, but redox and osmotic processes will recover asynchronously, demonstrating a decoupling between physical and biochemical restitution [104,105]. Delays in recovery due to oxidative stress will occur in mitochondrial fusion–fission dynamics; oxidative stress will damage microtubules that facilitate the transport of mitochondria, thereby creating fragmented thermodynamic domains with delayed equilibration [106]. In addition, photothermal stimulation of plasmonic nanorods at 808 nm provides complementary insights. The decay of the thermal signal defines the energy recovery half-life (τ^R^) and thermal conductivity (κ); in the inflamed cortex, τ^R^ is increased approximately six-fold and κ is decreased by half, demonstrating less rapid and spatially incoherent energy transfer [107,108]. Secondary acoustic waves produced by transient tissue expansion will also be detected via photoacoustic signals. In healthy tissue, these waves will be brief and harmonic; during inflammation, the waves will broaden, indicating the fractured fragmentation of thermomechanical coherence [109]. Collectively, the magnetothermal and photothermal assays define how efficiently energy “returns home,” the physical manifestation of neuroenergetic compliance [110].

### Dynamic Recovery Metrics: The Temporal Geometry of Reversibility

The recovery process following perturbation will unfold as a hierarchical temporal progression: mitochondrial redox normalization precedes astrocytic calcium oscillations, vascular dilatation, and cortical field re-entrainment [111]. In normal tissue, the recovery times for the redox (τredox), thermal (τthermo), vascular (τvaso), and electrical (τelec) domains will be near equal, suggesting an integration of feedback processes. Inflammation will abolish the equality of these times: redox recovery will slow, vascular overshoot will increase, and thermal relaxation will fall behind biochemical recovery [112,113]. The variability among these recovery times will define the Recovery Asymmetry Index (RAI), a quantitative measure of the desynchronization of the recovery processes. In a healthy cortex, RAI ≈ 0; in microglial activated areas, RAI > 0.4, denoting a lack of energetic synchronization [114]. At the microscopic level, the primary site of disruption is the astrocyte–vascular interface. Swollen astrocytic end-feet and diminished pericyte contraction will prolong the recovery of vasomotion and produce fluctuating patterns of alternately increasing and decreasing vasodilation/vasoconstriction that inject mechanical energy back into parenchymal tissue as microthermal turbulence [115]. Electrophysiological recordings will demonstrate delayed re-entrainment of field potentials: the phase delay between hemodynamic recovery and ionic recovery will widen, indicating a disconnection between the neural tissue and blood vessels and a slowing of the intrinsic “clock” of cortical coherence [116].

Repetitive perturbations will also elicit a second phenomenon, namely, perturbation hysteresis, the energetic “memory” of tissue. Unlike healthy tissue, where the recovery to equilibrium after each cycle is identical, inflamed areas will develop a cumulative deviation: temperature will dissipate more slowly, baseline redox potential will increase, and thermal noise amplitude will increase [117].

These effects result from conformational strain in the mitochondrial membrane and partial oxidation of respiratory cofactors, which leave molecules incapable of returning to their reduced state [118]. The per-cycle energy loss, ΔQ_h_, quantifies this hysteresis; if ΔQ_h_ > 10^−15^ J μm^−3^, recovery will be incomplete and signify the boundary between reversible and chronic injury [119]. Reactive astrocytes will further enhance the energetic memory through the reinforcement of their cytoskeleton, which will embed the signature of inflammation into the mechanical structure of the glia [120]. The slope of the fatigue curve—the rate of decline in recoverability—will mirror mechanical fatigue in metals, but here represents the biological fatigue of energy recovery [121]. Mathematical modeling extends these findings toward predictive biomarkers. Finite element thermodynamic and stochastic reaction–diffusion models will generate spatial distributions of the entropy diffusion coefficient (Ds) and energy restitution ratio (ERR) that describe the degree to which dissipated energy is recovered [122]. In the normal cortex, Ds ≈ 2.5 × 10^−7^ m^2^/s and ERR ≈ 0.9, signifying efficient energy recovery. In the initial stages of inflammation, ERR will decrease to approximately 0.6; in chronic states, it will be below 0.3, signifying uncontrolled energy diffusion. These changes will correlate with synaptic density, mitochondrial integrity, and cognitive performance [123].

Multidimensional PESP datasets analyzed via machine learning will identify three primary vectors—temporal coherence (coupling of τ values), energetic elasticity (ERR and κ), and entropic propagation (Ds and ΔQh)—that together form the Dynamic Thermodynamic Index (DTI). DTI will correlate with both histological and behavioral markers and will outperform traditional MRI/PET in detecting pre-symptomatic inflammatory changes, signaling a transition from biochemical to physical diagnostics [124]. Table 2 is designed to produce a representation of the synthesis of the modeling hierarchy from finite element thermodynamics to stochastic reaction–diffusion techniques.

Moreover, the future generations of diagnostic systems will not only measure but learn from these energetic reflexes. Implantable platforms marrying plasmonic heaters and UCNP thermosensors to NV quantum readouts could produce microthermal perturbations while at the same time interrogating the kinetic properties of the recovery mechanisms. Coupled with adaptive algorithms, these platforms would be capable of enabling material modification through the amplitude of pulse and time of stimulation on a real-time basis so as to guarantee allostasis, clearly independently affecting thermodynamic testing of neural tissue [131].

These closed-loop systems would serve potentially as dynamic barometers of resiliency. If τ^R^ is increasing or ERR is going down below some defined boundary, the system would identify the indicia of the initial fingerprint of the effects of thermodynamic fatigue—weeks prior to overt neurodegeneration. Such systems would be of de facto use for understanding of the diagnosis, and would serve for informing targeted intervention: fine-tuning of neuromodulation or nanoparticle modulation so as to be able to restore localized energetic indicia of symmetry [132].

The meaning of the definition of the resultant brain “health” of the perturbational energy–stress phenotyping means a change. It is not the static measure of function but that of the living physics of the process of recovery, the elegance with which the brain undergoes disturbance taking place in it and again returns to order. In a humble fashion of speaking about the brain, the objects have not been forced to disclose the subjection of such organs from the hand that has studied such a mechanism to be perceived as weak, but are now shown as its strength, hidden from view, the placid endurance of order in the environment of the perpetual thermal–dynamic disturbance [133].

Using the perturbation methods used in this research can lead to the possibility of converting the dynamic characteristics of the systems studied into data that can be used for diagnosis. All of the spatial and temporal patterns (recovery asymmetry, coherence decay, and entropy) observed during an energy–stress test are not only a representation of the physical properties being measured but are quantifiable measures of biological health, and when integrated at the molecular, vascular, and system levels they provide a single, identifiable thermodynamic fingerprint of the inflammatory brain. This conversion from kinetic response to quantifiable signature is what leads to the development of the new concept of thermodynamic biomarkers; where the stability of energy flow becomes a diagnostic measure of how disease progresses or does not progress and its associated level of resilience.

## 5. Thermodynamic Biomarkers of Neuroinflammation: Translating Energetic Disorder into Measurable Signatures

Neuroinflammation is an emergent phenomenon, not simply a molecular phenomenon. The salient events of neuroinflammation are not molecular, they are thermodynamic. Changes in coherence of heats and redox oscillations and mechanical compliance predate gross destruction of cells. The most salient future potential discovery of biomarkers is implicated in the ability to measure to high degrees of specificity these constraints, along with a feat of maximization of entropy itself as data. Thermodynamic biomarkers transform the non-measurable choreography of energetic disorder into an ordinality of measurable signatures, a metric of the physical coherence of the theory of a world of quantum reactions, cellular dynamics, and systemic metabolism [134].

### 5.1. Entropic Field Biomarkers: Imaging the Spatial Geometry of Disorder

Thermodynamic pathology is measurable through the imaging considerations of distorting heats and redox fluxes in remitted brain tissues. This realization has led to the advent of advanced magnetic resonance thermal imaging (MRT), photoacoustic tomography (PAT), and diffuse optical coherence imaging (DOCI), whereby it has become possible to employ three-dimensional mapping and reconstructive imaging of the entropic structures of the central nervous system at the millimeter scale [135].

Under physiological conditions the thermal fields are continuous gradients which ergodically model an energy flux. However, under conditions of neuroinflammation the continuity proceeds to fractalized microdomains whereby the freedoms of temperature and partial pressure of O_2_ present a scale invariant irregularity. The fractal dimension of heat (D_th_), which models this irregularity, is one index of thermodynamic pathology, whereby an increase from 1.2 to above 1.6 indicates a breaking down of the homogeneity of heat diffusion and locally an advent of thermodynamic fragmentation [136]. During neuroinflammation this fractal nature of the microdomains would indicate perivenular glial activation and mitochondrial clustering. This indicates that D_th_ is a useful non-invasive marker of thermodynamic fragmentation. Concomitant PAT and DOCI analysis shows that the oscillations of vascular oxygenation exhibit decoupling of normal phase rotation with perfusion pressure, i.e., oxygen–pressure phase drift (ΔφO_2_–P). This is present in the inflammatory areas, where ΔφO_2_–P is approaching π/2, being almost complete decoupling of the metabolic needs and vascular supply. By co-registering D_th_ and ΔφO_2_–P, a consistent spatial overlap is shown: where heat and oxygen viability vanish are co-located those same places where the density of entropy approaches its maximum [137,138].

Magnetothermal resonance elastography (mTRE) locates the mechanical dimension, expressing the relative physical interaction of thermal changes with the viscosity of the tissue. The coupling coefficient (χ_th_–μ), expressed as the conversion efficiency of thermal to mechanical energy, reduces by as much as 50% during inflammation. Thus D_th_, ΔφO_2_–P, and χ_th_–μ define a triplet of biomarkers of field entropy, being indices of physical image diagnosis, which gives geometrically the disorder of entropic energy before appreciable structural damage is established [139].

### 5.2. Molecular Correlates of Energetic Instability: Biofluid Thermodynamics

This energetic field disequilibrium establishes in cohered reflections a measurable seral signature upon circulating molecules, as the body fluids themselves show the general systemic diffusion of entropic orders. The oscillome vintage of plasma redox, defined by the cyclical variation in the Nad^+^/NadH and GSH/GSSG energy ratios, represents the systemic rhythm of energy exchange. Neuroinflammatory pathology causes the remoteness of amplitude and temporal coherence of the redox oscillation, the phenomenon being designated redox decoherence. The coherence index (C_re_d_ox_), which piecewise engenders spectral decomposition of the redox time series, is inversely correlated with the cortical entropy flux measured by thermometrical imaging [140].

The cerebrospinal fluid (CSF) simultaneously shows an inclination to surge forth a higher degree of temperature protecting proteins (thermoprotective), HSP70, HSP90, and mitochondrial chaperones such as TRAP1. Molecular chaperones can act as local entropic suppressors by binding up the mistaken proteins and so inhibiting the dispersal of energies into non-energetic vibratory states. That is, the sum of the ratio HSP to the amount of proteins, HSPm/TP, can be considered broadly a measure of the molecular elasticity coefficient (Emol) of the given object; a high Emol means temporary hardness of the object, while chronic catabolism or irreversible energetic fatigue of the given object means chronic energetical fatigue of the object in question [141]. The carriers of the energetic status on a nanoscale are given by the extracellular vesicles (EVs). The EVs produced in the neuroinflammatory microenvironment show an increased lipid oxidation, a different thermotropic function, and an increased quantity of smaller fragments of mitochondrial RNA, which indicate the ROS energetic metabolism pathway. Using nanoscale surface plasmon resonance (SPR) spectroscopy, the ratio of the plasmonic absorption Rpl of the single EVs can be measured, and a meter of the energetic absorption on the nanoscale will then be produced; the larger the Rpl, the larger the internal enthalpy unit volume, which means that the dense energy is also measurable as a molecular quality [142]. The biochemical markers that form these biofluid signatures connect to the established body of work related to other neuroinflammation biomarkers (e.g., cytokines, chemokines, and microglia activation tracers) that currently support the majority of diagnostic methods for detecting and quantifying the biochemical manifestations of inflammatory processes; however, they do not provide insight into the biochemical representations of the fundamental energetic mechanisms driving those inflammatory processes [143]. The biochemical parameters described above (i.e., redox coherence indices, molecular elasticity coefficients, and nanoscale plasmonic absorption ratios) extend the conventional biochemical approach to diagnosing and quantifying the biochemical manifestations of inflammation by incorporating measurable descriptions of changes in the biochemical organization of energy [144]. Therefore, the biochemical framework used for diagnosing and quantifying biochemical manifestations of inflammation, and the new thermodynamic framework for diagnosing and quantifying the biochemical manifestation of inflammation as it relates to changes in the biochemical organization of energy, are now connected by a common continuum in which the biochemical concentrations and biochemical organization of energy are two complimentary dimensions of the same physiological process [145].

### 5.3. Nanothermodynamic Biosensing: Capturing the Invisible Flux

In order that it might be possible to detect the earliest and the most delicate kinds of eruption of energetic “irregularities” that are the forerunners of inflammatory activity of the organism, biosensors composed of nanoparticles, which will be able to pick up thermal and redox gradients present at the same time, were devised. These sensors will bear the plasmoids that submit the fluctuations of all sciences to quantitative lights or measurable electrical signals, hence trying to integrate the nanoscale physics of inflammation into our macroscale techniques of biomedical diagnosis. A fine example of the concomitant function of the two in question is given in the Janus thermoredox nanoparticles, which have a lanthanide up-conversion core segregated from a gold–palladium catalytic skin. In the emission spectra, temperature-sensitive green bands and redox-sensitive blue bands will be seen; the sum of intensities being the index of Nano-Entropy (NEI) [146]. The NEI grows exponentially when increased with the local imbalance of reaction kinetics possessing exothermic thermodynamics or endothermic of the given state of affairs, which gives an increase in the real chance of exact quantitative determination of an energic or theoretical kinetic irregularity of equilibrium in the local volume of the microvolumes of the CSF or in the plasma [147]. The thermoelectric nanowires on the flexible microchip measure temperature noise using the Seebeck Effect. In neuroinflammatory fluids there is a chaotic thermodynamic randomization rendered in electrical signals with statistically increased spectral entropy. The Entropy Power Coefficient (EPC), defined in terms of the frequency/amplitude distribution of this electrical noise, is the first electrical measure of biological entropy production. The NEI-EPC profiles differentiate active from resolving inflammation with greater sensitivity than the presently available cytokine assays [148].

In another invention, biodegradable nanocalorimetric implants have been fabricated which consist of hydrogels loaded with lanthanide complexes, the fluorescent lifetime of which imparts information about the thermal and the redox potential. When these implants are inserted in perivascular tissues a continuous readout is obtained of the local rate of entropy production (σ_t_). Longitudinal recordings permit an increase in σ_t_ some days in advance of the activation of the microglia and thus serve as an ultra-early predictive biomarker [149].

### 5.4. AI-Integrated Thermodynamic Pattern Recognition: Energetic Connectomics

The complexity of the thermodynamic biomarker requires that the analytical models integrate the information from diverse modalities and scales. Artificial intelligence algorithms trained in multimodal data bases, incorporating thermometry, spectroscopy, measurement of bio-signals, and imaging of the tissues, extends to the classification of the energetic state of the neural tissues with admirable fidelity [150].

The machine learning operative teases out three principal axes of discrimination for classification:

- Redox–thermal coupling (phase coherence between NADH oscillations and variance of temperature);
- Mechanical–viscous opposition (integration of Θ_v_ and χ_th_-μ);
- Entropy diffusion symmetry (correlation of D_th_ and σ_t_ spatially).

These parameters express a relationship representative of a coordinate system termed the Thermodynamic State Vector (TSV). In this high-dimensional space, healthy brains lie near the low-entropy origin while inflamed systems follow paths of increasing asymmetry and diffusion anisotropy [151].

Network analysis adds another layer of interpretation. By making a thermodynamic connectome from voxel-wise covariance of entropy flux, it can be seen how the disorder of energy propagates through the structural conduits of the system. Healthy brains exhibit a small-world topology—short, efficient paths of energetic equilibria [152]. Neuroinflammation obliterates this modularity: entropy propagation becomes isotropic and percolative, in keeping with the loss of topological control. Proposed from this analysis are two new connectomic metrics:

- Connectomic Entropy Density (CED)—the global rate of entropy accumulation per edge of network;
- Thermodynamic Clustering Coefficient (TCC)—the degree to which energy fluctuations are retained in a local state;
- Simulation of CED elevation and TCC collapse predicts joint progression toward irreversible energetic decoherence.

The reliability of the AI-driven thermodynamic classifiers has been examined using systematic cross-validation and perturbation testing for consistency in their predictions. The multimodal dataset (nanothermometric, spectroscopic, and hemodynamic) was separated into two cohorts, an independent training cohort and an independent validation cohort. Five-fold cross-validation was used to assess the stability of the models [88]. Gradient-based attributions and permutation analysis revealed that τ^R^, σ_t_, and D_th_ are the most important features associated with the predictive accuracy of the models. Using feature reconstruction, the AI-models can also reproduce the thermodynamic trajectory of unseen datasets. Furthermore, this trajectory is consistent in terms of the clustering of energetic phenotypes within the Thermodynamic State Vector space (mean deviation <5%) [153]. The results of the above validation methods demonstrate that the AI models identify physical relationships between variables as opposed to identifying spurious relationships between variables. This is further evidence that the energetic connectomics is a reliable method for diagnosis, as it is a reproducible diagnostic framework [154].

### 5.5. The Thermodynamic Biomarker Atlas: A Unified Framework for Energetic Phenotyping

As individual measurements give way to coherent patterns, a wider vision emerges—the Thermodynamic Biomarker Atlas (TBA). This atlas serves as a conceptual and empirical repository of energy-based indices whereby each state of brain energy is placed within a multidimensional thermodynamic continuum [155].

The TBA has four levels of hierarchical strata:

1. Field-level meteorics: D_th_, ΔφO_2_–P, χ_th_–μ—geometric descriptors associating with the distribution of energy;
2. Molecular endpoint indices: C_re_d_ox_, E_mol_, R_pl_—biochemical correlates of redox–thermal adaptation;
3. Nanoscopic readouts: τ^R^, Θ_v_, NEI, EPC, σ_t_—direct physical measures of micro-scale disorder;
4. Network variables: TSV, CED, TCC—system-level descriptors of entropy propagation.

When the variables are mated in a relationship, they form a four-dimensional energetic phenotype that may be visualized as a dynamic surface which undergoes time changes. Early information corresponds to slight elevation of D_th_ and C_re_d_ox_ reduction, whilst advanced disease is manifest in complete topological collapse of network energy flow. The TBA thus provides a unified diagnostic ontology where thermodynamic imbalance becomes quantifiable, comparable, and reversible [156].

Ultimately, thermodynamic biomarkers shift the definition of pathology itself. Rather than classifying disease by structural loss or molecular accumulation, they classify it in terms of the failure of coherence, that is, the point at which biological energy ceases to self-organize. By anchoring diagnosis in universal principles of the physical universe, this framework aims toward transcendence beyond the boundaries of disease per se and places neuroinflammation, degeneration, and repair together in a common energetic language [157].

If pathology can be viewed as an identifiable failure of coherence, which is measurable, then restoration of that coherence via physical methods would be the goal of therapy. Likewise, the principles that allow for the measurement of entropy and energetic asymmetry can also direct nanoscale interventions to restore order to the flow of energy. Diagnostic thermodynamics will provide information about both where and how coherence is lost. Nanothermodynamic approaches will provide the tools to rebuild coherence at the nanoscale using nanoscale catalysts, optical fields, and adaptive feedback to restore the rhythmic equilibrium between heat, charge, and flow. Therefore, the transition from biomarkers to intervention is not a paradigm shift; it is rather the completion of that paradigm—the transformation of understanding of energy into energetic healing.

## 6. Nanothermodynamic Interventions: Restoring Energetic Coherence in Neuroinflammation

Neuroinflammation is an example of a physical failure of self-organization of the nervous system—and therefore of thermodynamics—which represents a breakdown in coherent energy flow across mitochondria, glia, and vasculature. Thus, nanothermodynamic intervention acts directly upon those physical failure modes—it will rebalance heat and redox fluxes, synchronize slow bioenergetic rhythms, and re-establish glial–vascular conductivity, utilizing catalytic nanosystems and adaptive fields to decrease the production of entropy and restore reversibility [158].

### 6.1. Rebalancing Heat and Redox Fluxes Utilizing Hybrid Nanoparticles

When inflammation disrupts the integration of thermal, redox, and ionic currents, nanothermodynamics can be utilized to treat neuroinflammatory conditions. Thermoplastic nanorods (Au or Ti doped, 808–980 nm NIR peaks) are capable of delivering very small amounts of heat to the mitochondria in astrocytes, resulting in very slight increases in temperature (less than one degree Celsius), thereby gently reducing micro gradients across astrocytic syncytia and perivascular niches—we refer to this process as thermal pacing. Thermal pacing is necessary for coherent oscillations of redox and thermal currents [159]. Simultaneously, catalytic clusters (such as CeO_2_ and Pt-Ir) function as reversible electron sinks (Ce^3+^/Ce^4+^; Pt^2+^/Pt^4+^) when conjugated to mitochondrial membranes via triphenylphosphonium or cardiolipin analogues. These clusters stabilize NADH/NAD+ cycling in the exact location where electron and heat flux merge—the cristae [160].

Thus, a combination of both functions may be achieved utilizing Janus thermoredox nanoparticles, which have a photothermal-absorbing surface and a catalytic surface. The photothermal surface has been designed to absorb near-infrared radiation and to produce a gentle heat stimulus, while the catalytic surface contains a reversible redox couple (such as Ce^3+^/Ce^4+^, or Pt^2+^/Pt^4+^) to facilitate the exchange of electrons. Once infused into an inflamed network, the nanoparticles behave as local entropy correctors; they narrow the dispersion of Δψm, lower τ^R^ by approximately sixty percent, and facilitate the reappearance of coherent oscillations of redox and thermal currents within minutes of infusion—consistent with coherence percolation through gap-junction-coupled glial lattices and subsequent cortical resynchronization [161,162,163].

### 6.2. Entrainment of Temporal Oscillations Using Adaptive Fields

For systemic level coherence, entrainment (not stimulation) is required. The application of low frequency magnetic interference (LFMI) applies 0.5–3 mT magnetic fields at frequencies of 0.1–1 Hz—aligned to mitochondrial redox envelopes—to lower stochastic tunneling noise at complex IV and restore phase alignment of NADH waveforms; imaging readouts demonstrate re-coherence of vasomotion and ~40% variance reductions in heat/metabolic potential after LFMI [164,165]. Photobiomodulation (PBM) at wavelengths of 810–860 nm elicits NO production from cytochrome-c-oxidase, improves oxidative flux and ATP production; the net results are decreased ΔσO_2_ and increased ERR, i.e., increased thermodynamic efficiency [166,167]. Quantum field-alignment protocols (picotesla oscillatory fields) modify H-bond networks in the mitochondrial water shell and modulate H^+^ transfer through the F_0_ channel, decreasing entropy dissipation without metabolic cost—an information-based correction of phase order [168,169].

Taken together, LFMI, PBM, and quantum field alignment restore hierarchical synchronization of mitochondrial, vascular, and slow cortical oscillations—the thermodynamic basis for functional reversibility [170].

### 6.3. Reintegrate Glial-Vascular Conductivity

Gliovascular disorganization is the major lesion in the thermodynamics of inflammation. Restoration of AQP4 polarity at the astrocytic end-feet restores perivascular convection of heat and solutes; AQP4 clustering peptides or mRNA stabilizing agents increase perivascular heat flux (qp _v_) and decrease local entropy density (σt) [171,172,173]. On the other hand, restoration of pericyte mechanotransduction restores vasomotor compliance that is otherwise disrupted by inflammation and NO signaling disruption [174].

Mechanosensitive nanocapsules that release vasodilators under shear stress restore pressure–oxygenation phase coupling, whereas Cx43 reactivation (via mimetics or gene editing) restores contact between astrocytes to form conductive lattices. The result is a decrease in the entropy-diffusion coefficient (Ds), the reappearance of synchronized Ca^2+^ waves, and measurable lengthening of energetic coherence in nanothermographic maps [175,176].

### 6.4. Closed-Loop Nanotherapeutics: Self-Regulating Thermodynamic Control

An advanced method for treating neuroinflammatory conditions is a self-regulating thermodynamic prosthesis, which includes the components of sensing, modeling, and actuating in vivo [177]:

- Quantum sensing layer: NV-diamond centers continuously measure sub-mK thermal and magnetochemical readings (τ^R^, σ_t_) [75];
- Cognitive controller: neuromorphic or external AI models estimate deviations from equilibrium and select minimum energy corrections [178];
- Actuation module: plasmonic heaters and redox catalysts deliver millisecond-scale localized stimuli [179].

Computer simulations and bench-top models demonstrate that these systems can maintain tissue temperatures within ±0.05 °C and redox ratios within ±5% of baseline throughout chronic inflammatory cycles, thereby effectively externalizing a sliver of homeostasis to maintain energetic coherence [180,181].

### 6.5. Therapeutic Endpoint: Thermodynamic Recovery

**Figure 3:**
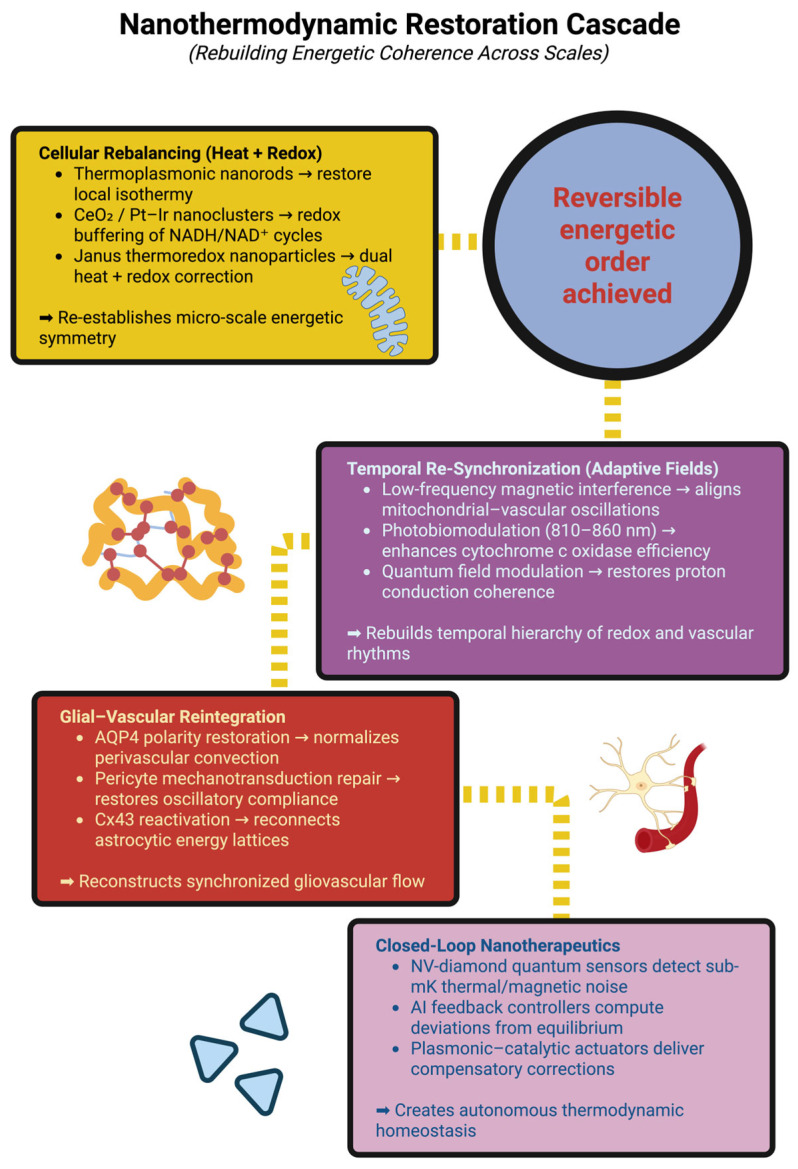
Nanothermodynamic restoration cascade: rebuilding energetic coherence across scales. This schematic illustrates the hierarchical sequence of interventions designed to restore energetic order in neuroinflammation. The process begins with cellular rebalancing, where thermoplasmonic nanorods and redox-active nanoclusters re-establish local thermal and redox symmetry. Temporal re-synchronization follows through adaptive field modulation—low-frequency magnetic interference, photobiomodulation, and quantum field alignment—realigning mitochondrial, vascular, and cortical rhythms. Glial–vascular reintegration repairs the astrocyte–perivascular interface via AQP4 polarity restoration, pericyte compliance recovery, and Cx43-mediated coupling, rebuilding the pathways for coherent energy flow. Closed-loop nanotherapeutics integrate quantum sensing, AI feedback, and nanoscale actuation to continuously correct deviations from equilibrium. Collectively, these stages converge toward reversible energetic order, marking the re-establishment of systemic thermodynamic homeostasis and coherence in the inflamed brain.

Regardless of modality, the target of therapy is coherence; namely, restoration of phase-locking between mitochondrial redox cycles and gliovascular oscillations, and the recovery of low-frequency coupling among temperature, blood flow, and neural activity [182]. This corresponds to clinically observable decreases in entropy flux, increases in ERR, and the return of low-frequency coupling among temperature, blood flow, and neural activity. Therefore, nanothermodynamic therapy defines “cure” as the renewal of energetic order rather than merely structural restitution—the inflamed brain re-learns its rhythmic behavior and converts disorder into controlled fluctuations, thus maintaining the possibility of reversible dynamics [183]. It is expected, as entailed in the illustrated schematic in Figure 3, that the cascade of nanothermodynamic restorative is produced, thus establishing the coherence of energies of functional integrity across biological scales.

## 7. Translational Integration: From Thermodynamic Metrics to Predictive Neurotherapeutics

The translational frontier of neuroenergetics lies at the conjunct junction of classical neuroscience and physics, which interfaces between molecular signaling and the thermodynamic laws of the flow of energies/bodies. It must be considered thermodynamically that the causative agents of neuroinflammation are not solely an immune cascade but a lack of coherence in respect of the way in which the brain utilizes neo-recycling of energy through the hierarchical networks it commands. Thus, the new emerging scope of Translational Neurothermodynamics is to measure this loss, strike predictive outcomes, and create the symmetry re-establishment prior to structural degeneration. Ultimately, by the use of traditionalisms of the molecular–physiological–imaging–nanotechnology of sensory modality, discovered in principle in one unified energetic model, the inflamed brain is seen not as a static lesion but as a dynamic measurable and reversible process [184].

### 7.1. Multiscale Thermodynamic Modeling of the Inflamed Brain

The brain, as a multi-staged energy field, is kept integrated through the continuous recycling of energies of production by molecular heat generations, vascular carrying-through, and glia–neural simple affectivity. It is the incoherence of transmission at these different levels of energy that is so negatively impacted by neuroinflammation, allowing non-sustainability. Modeling of this coadunation necessitates the conjunction of micro-scale entropic graduation to macro-scale structural failure [185]. At the cellular scale the mitochondria may be considered as quantized oscillators of energy cum inversion producing energetic masses, utilizing redox-potential structured modalities in order to flow in structured modalities. The state of inflammatory failure of the electron transport system leads to loss of energetic coupling efficiency and leads therefore to increased production in the locus of heat. In calorimetric studies it is discovered that in the cortical neuron there is an increase in heat flux (σ_t_), where the capability of oxidized-complex IV is lower; hence this leads to spontaneous micro-fluctuations in temperature and this destroys calcium waves in neurophyl-minding astrocytes. These waves transmit through gap-junction neuro-phylness networks as heat/ionic sterility, and hence transport a coherent organismal wave experience into noise and stochasticity of fields [186].

At the glial–vascular interface, astrocytic end-feet and pericytes are normally optimized for thermal buffering, converting the metabolic heat of the brain into periodic vascular pulsations under regulated control conditions. Whenever the inflammation of the underlying normal physiology disturbs the localization of aquaporin-4 or the contractility of pericytes, this leads to a decoupling of the flow of heat and oxygen from the neural need. This decoupling is made apparent in measurable terms in relation to changes in the oxygen-pressure phase shift (ΔφO_2_–P), this being a parameter which is linearly proportional to local entropy density. Computational models show that when ΔφO_2_–P > 0.4π relative to normality, the vascular oscillations lose their resonance with neuronal activity, leading to regional hypometabolism relative to normality, even although blood flow is intact [187].

At the level of neural networks, the energy carbon transfer follows topologic rather than diffusional rules. Graph-theoretic constructions of the thermodynamic fields in neocortical tissue show that healthy brains operate under a “small world” topological structure with high clustering and long-range link parameters. The normal topology is damaged with neuronal inflammation: entropic diffusion becomes isotropic, and the modular nature of the network collapses. The coherence length (λc) of thermal correlation falls below 2 mm, indicating the transition from ordered wave propagation to chaotic dissipation. The recovery of λc by re-synchronization produced either by nanothermodynamic or neurological means becomes a measurable end-point of productive therapy [188].

This gaming at various levels indicates that neuroinflammation is fundamentally a phase disorder of energy transformation, whose repair is a recoupling of the symmetry of coupling at the physiological molecular, glial, and vascular levels [134].

### 7.2. Personalized Entropy Profiling: A Dynamic Framework for Predictive Medicine

To translate the insights of physical processes into the decisions to be taken on the level of clinical management, a dynamic therapy process, Personalized Entropy Profiling (PEP), has been evolved. Personalized Entropy Profiling brings together thermolabile information from a variety of modalities into an individual energy trajectory which flows in time, and permits a diagnostic measure of the stability of the economy of the brain in real time. The model describes a thermodynamic phenotypic vector (TPV) of six major parameters; entropy production (σ), coherence co-efficient (κ), capacity for restitution (ρ), coherence length (λc), relaxation time (τ^R^), and energy restitution ratio (ERR) [189]. These parameters exist in a continuous thermodynamic manifold (M_th_)—a poly-dimensional phase space describing the balance of order and dissipation in the brain. In the earlier stages of inflammation, the TPV oscillates around an equilibrium point and is a reversible oscillation. As the pathology increases in severity, however, so does the drift of the TPV to high-σ, low-κ attractor basins, which are an instance of an irreversible process of entropy production [190].

**Figure 4:**
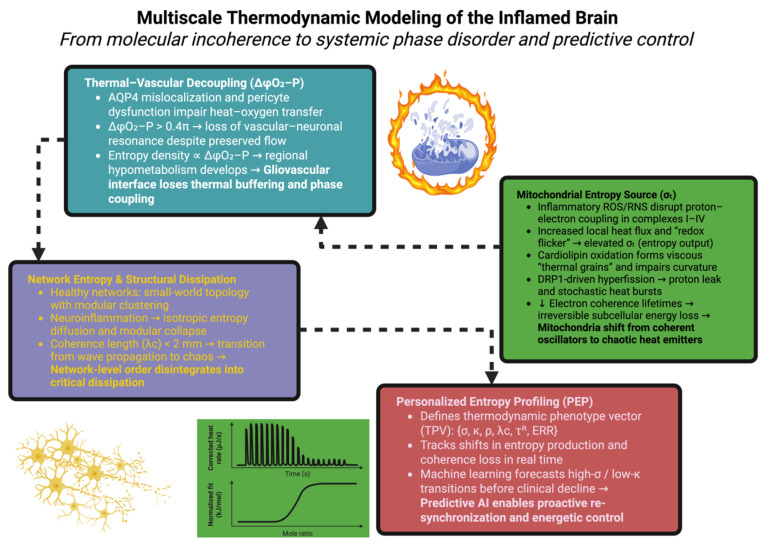
Multiscale thermodynamic framework illustrating how inflammation disrupts energetic coherence from mitochondrial oscillations to systemic entropy profiling. Each level—molecular, vascular, network, and computational—represents a distinct but interconnected domain of dissipation and potential therapeutic recoupling.

By the application of machine learning algorithms that are applied to longitudinal PEP data streams, these transitions can be predicted by D- or R- days in front of actual overt clinical ill-health. Entropy time series analysis, for example, using recurrent neural networks, may detect energy dephasing events, leading to cognitive decline in neurodegenerative models. In such paradigms means-initiatives of therapeutic interventions become proactive rather than reactive, and are mapped to prevent the system being dumped into low-σ, high-κ states prior to geometric collapse. Thus neuroinflammatory disease itself becomes a navigable trajectory in energetic phase space, and is able to be kept in a state of rest by ongoing re-synchronization [191]. The schematic in Figure 4 intends to show how neuroinflammation occurs through a multi-scale disruption in energetic coherence, which ties molecular heat production to systemic phase transition instability. This figure illustrates how inflammation will transform mitochondria from coherent redox oscillators that are coherent into stochastic heat sources, decouple the thermoregulatory and vascular feedback loops at the gliovascular interface, and collapse the network topological structure into chaotic dissipation.

### 7.3. Closed-Loop Neuroenergetic Therapies: Controlling Entropy in Real Time

The introduction of nanotechnology, quantum sensing, and AI modeling now allows for the effects of the closed-loop energetic regime to be carried out; to measure and manipulate the dynamic entropy of neural tissue. Implantable thermodynamic operational venues comprise nanoscale sensors, adaptive controllers, and catalytic actuators, all incorporated into flexible micro-architectures of the nitrogen-vacancy (NV) diamond sensors, which detect sub-millikelvin fluctuations in temperature and fluctuations in magnetic noise of nanotesla order, thus providing feedback in real time of τ^R^ and σ_t_ in cortical or perivascular compartments [192]. These data feed into neuromorphic processors, which via their effect on thermodynamically filtering detect deviations from baselines of coherence [193].

Once the entropy exceeds a predetermined preset, responsive proportions of either redox activity or thermo-plasmonic nanoparticles are scaffold released, which respond to local gradients of either chemical variation or thermal variation [194].

The systemic feedback adaptations in preclinical models afford redox stability and homogeneity of cortical thermal homeostasis under inflammatory stress, obliterating over sixty percent of entropy thrown off due to variational loading of communication. Meanwhile, back-effects occur in the restored functional energetic order of the field of neuroimaging in the cortex, of phase coherence of the distribution of temperature generated in the cortex, and the pulsatility of the vascularity-re-established energetic functional order [195].

Similarly, parallel-interface biotechnology, in systems of tele-thermodynamic bio-compatibility, externally wearable and implantable, enable the sentient data of the energetic state to be continuously given to Gn AI networks, with capacity to self-adjust. Thus, skin surface temperature fractality, diffuse properties of heat diffusion in microvessels, and redox potential are measures peripheral to measures of energetic state, coupled centrally with a systemic effect on coherence of entropy states. Again these measures of continuous data streams of energetic measures allow cloud-based recalibration of either stimulus parameters or nanomaterial doses, to move us from the interventional episodic energetic medicine into the age of adaptive energetic medicine, where homeostasis is obtained through continuous data feedback systems rather than episodic interventional work [196].

### 7.4. Network Entropy as a Quantitative Marker of Recovery Potential

Entropy itself as a measure of disorder is therefore a measure of capacity for adaptation to life in general. The neural entropy of the network distribution itself is a measure of the capacity of the cortical systems of the distribution of energy from central generative to peripheral functions, without failure or collapse in structure. High states of entropy are random dissipations of energy. The optimal states of entropy inclusive imply structural peaked phasic flexible distributed dynamic structural entities [197].

Two measures of subtlety evolving from the structure of the network itself, Connectomic Entropy Density (CED) and Thermodynamic Clustering Coefficient (TCC), have been pushed out as the most sensitive parameters. In healthy networks of functions of networks, CED is maintained at values of less than 0.3, and TCC does not drop below 0.6, and hovers around, therefore, energies of distribution of modularity of inter-tissular dynamicity. Neuroinflammation increases CED to approximately 0.8 and reduces TCC to below 0.4, meaning total entropy communication [198].

Nanothermodynamic and field-directed interventional modulation can significantly change these indices: entropy diffusion is restricted in days of adaptive supervision factors with renewal of TCC moving back toward physiologic levels. Connectomic imaging analyses determine how small-world topology is rediscovered in conjunction with behavioral recovery—due to the fact that energetic coherence underlies functional recovery [199].

AI programming thermodynamic reinforcement learning can effectuate a recursivity of this conceptual design to specifically lower social energy cost nodes—known as entropic keystones—whose modulation would effectuate maximal systemic stability. These are lodged at of deepest cortex and thalamic relay hub loci, thereby serving entry at specific removement neuromodulations directed in task-specific terms with recovery of energetic hierarchies disordered whence remote inflammation occurs [200].

### 7.5. Toward Predictive Energetic Medicine: The Future of Translational Neurothermodynamics

The final vision of which is appearing out of this integrational framework is that of predictive energetic medicine—that discipline wherein disorder is to be known as measurable entropic imbalance, and healing is to be arrived at via intelligible restoration of coherence [201].

Future clinical platforms are to be the conjunction of magnetic resonance thermography quantum thermometry–extracellular vesicle spectroscopy and an AI medium into one integrated energetic dashboard. This will visualize in real time the evolution of entropy coherence and restitution within the patient’s brain. The clinicians are not to treat symptoms but only stabilize the trajectory—so-called—before irreversible phase transition appears prior to energy mediation occurring [202].

Such an approach is of course not going to be limited to inflammatory events. It is possible that the same thermodynamic principles are necessary to inform the degenerative–traumatic–age-induced cognitive decline underway of either case with a common factor of gradually lost energy symmetry. The estuarine coherence regained is of mitochondrial–glial–vascular networks; within the organic brain there must lie plasticity, not now by suppression, but instantly via reordering now energy economy is possibly reinstated [203].

In conclusion, the future of the neurosciences may not lie in the elucidation as to how the brain functions to organize storage of memory, but how it is supported on its oscillatory and yet reversible fields of energy-flow—the primal sine qua non of learning-repair and consciousness itself. Predictive energetic medicine will make a matter of this definitively comprehensible, controllable, and ultimately preservable over a time span.

The goal of this framework is for it to be a total physics of healing; however, there are also contextual limitations to this framework’s comprehensiveness. The biological variability of individual differences—including sex-based differences in mitochondrial energy efficiency, redox flux modulated by hormones, and glial thermodynamic response—may affect the stability of energetic coherence among different individuals and alter the entropy dynamics among individuals. Additionally, the current analysis has been based largely upon preclinical studies utilizing model systems. Therefore, caution should be taken when translating these findings to the study of human neuroenergetics. Although the models used in this analysis have provided mechanistic insight into the process of thermogenesis, they cannot currently fully describe the temporal plasticity or the environmental influences that modulate the thermodynamics of living humans. Therefore, future studies using in vivo nanothermometry, with both male and female subjects (sex-balanced cohorts), and AI-assisted energetic mapping of neural tissue will be necessary to confirm the validity of this framework and to define the physiological boundaries within which predictive energetic medicine can be safely and widely applied.

### 7.6. Future Directions and Gaps in Knowledge

Although Translational Neurothermodynamics presents a single physical law that can link all molecular, energetic, and systemic process together, there are many barriers that need to be overcome before it becomes applicable to human health care. The first barrier is the lack of standards for experimental design. The currently available nanothermometric and quantum-sensing approaches have varying levels of calibration accuracy, they penetrate different distances into tissues, and they provide measurements over varying time scales; therefore, to create a common platform that would allow studies to compare results and to reproduce findings, a standardized method for measuring entropy production, coherence length, and relaxation dynamics must be developed [204]. A second major area that has yet to be addressed is the challenge of integrating data across multiple scales of organization. At present, the relationship between the loss of quantum coherence in mitochondria and the increase in macroscopic entropy in the cortex remains largely speculative. Addressing this problem will require development of hybrid models that can integrate stochastic energy fluctuations at the molecular level with ensemble-level behaviors that can be measured using imaging and spectroscopy techniques [205].

To go further than simply classifying energetic stability as either stable or unstable requires machine learning systems to move from classification to true causal inference so that predictive models can predict when energetic instability precedes structural damage [206]. In addition, ML systems must be transparent regarding how features were selected for analysis and how well their predictions hold up longitudinally across species and disease types to establish credibility. From a translational perspective, there is much work to do before these concepts become clinically relevant. While it is possible to measure entropy and energetic coherence in preclinical preparations, there is very little evidence of measurement of humans directly. Therefore, longitudinal multimodal studies are required to determine if restoration of energetic coherence relates to recovery of cognitive function [207].

There is still an important philosophical and multidisciplinary gap between the molecular language of medicine and the physical laws that govern open dissipative systems. Realistic understanding of neuroinflammation will depend on bridging the empirical precision of neuroscience with the predictive power of thermodynamics. Closing these gaps will change the conceptual basis of brain disease from being a static lesion to being a dynamic balance of energy; ultimately, from a condition where pathology is irreparable to a condition where loss of coherence is reversible [208].

## 8. Conclusions: The Reversible Brain—Toward a Thermodynamic Paradigm of Neurorestoration

Over the centuries in neuroscience, the understanding of the brain has advanced through anatomy, chemistry, and electricity; but below all of these there runs a fundamental substratum, a physics of organization, a ceaseless agitation of order and entropy which makes biological intelligence possible. Neuroinflammation, considered from this standpoint, is not merely a pathology of molecules, but a dissymmetry of energy and information. Its remedy must not, therefore, solely depend on pharmacology or betterment of genetic function, but must arise from a restoration of that coherence without which life cannot sustain its delicate thermodynamic balance.

The preceding sections have attempted to trace how this coherence might be measured, modeled and, if possible, restored. The concept of thermodynamic biomarkers changes the significance of temperature, redox potential, and molecular vibration into quantitative definitions of brain health, and reveals the early signs of malfunctioning long before structural injury takes place. Nanothermodynamic intervention extends the field into therapeutics, restoring dis-symmetries of energy, coordinating biological rhythms, and reconstructing the brain’s capacity for reversible modulation. In conjunction, these describe a new line of continuity between diagnosis and reparation, in which measurement and modulation become but two methods of expressing the same physical law.

What emerges is not a new molecular neuroscience, but a more profound background to it, an integrative science in which physics, biology, and computation arrive at a convergence in the notion of energetic coherence. The brain is thus presented as a dynamic system, permanently in the process of negotiating with entropy. Identity maintains itself, not by resisting disorder, but by forming it into patterned changes in its rhythm—oscillations, rhythms, and cycles, which register the stability of identity through change. Disease arises when this rhythm loses its symmetry; restoration is the effect when the power of re-synchronization reappears. The application of these reflections to therapeutics marks the birth of prognostic energetic medicine—a therapeutics in which entropy becomes a “sign of life.” The thermodynamic charts operational and in real time, constructed from quantum sensors and adaptable algorithms, will ere long enable the physician to visualize the energy flow through the elements of neural tissue as easily and vividly as the flow of blood through vessels. Therapy is no more a systaltic effort to restore stability to a system, but an inceptive effort to restore its resonance with itself. Nanoparticles, fields, and biologically informed algorithms will act no longer as agents from without, but as the extensions of homeostasis—as instruments that listen to the brain’s internal music, and help it to find its lost tune. This point of view modifies the conception of restoration. The aim is not to restore a previous state of things, but to reintroduce the possibility of reversibility—the possibility of fluctuation, adaptation, and return to order occurring in a system. This is indicative of the fact that chronic neuroinflammation, organic deterioration, intellectual poverty, or some phases of organic degeneration are not to be accepted as irreversible phases of decay, but as reversible phases of energetic disorganization. In restoring the physical preconditions for synchronization—thermal uniformity, redox equilibrium, and coherent vascular pulsation—we may reawaken the self-organizing principles that the brain naturally applies for the purposes of repair. The larger implication goes beyond disease: if energetic coherence conditions the parameters of learning, consciousness, and resilience, then such understanding opens a new language for cognition itself—for neural computation may ultimately be seen as entropy management—a dynamic of optimal energy flow, governed by the laws of physics. Every thought, every memory, every adaptation may be therein seen as a local triumph over despair—a transient crystallization of order in the vast thermodynamic field which sustains the self. In closing, this work does not claim to unravel the complex intricacies of the brain, but to suggest a new stand-point from which its unity once more becomes manifest. In attempting to portray neuroinflammation as a thermodynamic phenomenon, we have endeavored to show the common thread uniting molecular fluctuations, vascular uncoupling, glial decoupling, and network collapse—all manifestations of disordered energy. We suggest that the way onward lies not in greater specialization, but in a synthesis—namely, of molecular specificity and physical universality.

This paper proposes to offer more than a mere framework—it seeks an invitation. The invitation is to regard the brain not as an organ which consumes energy, but one which produces coherence. To study its pathologies not as the failures of the embodiment, but as aberrations of timing, temperature, and flow. And to envisage a new science of neurology where therapy becomes factually identical to calibration—where the clinician and the physicist, the biologist and the engineer, collaborate to preserve the reversible rhythm of the living mind. The true revolution in neurology may not come about through the discovery of new molecules, but through the rediscovery of an ancient truth: that life continues because it knows how to employ energy without itself being lost. The brain, because it is the organ’s highest growth, may be teaching us the same lesson, that the essence of intelligence is not complexity, but coherence.

## Figures and Tables

**Table 1:** Multiscale thermodynamic mapping of neuroinflammatory energy flow. Each physical tier—from femtosecond redox decoherence to macroscopic perfusion flattening—contributes a distinct increase in entropy production (σ) and a measurable loss of temporal or spatial coherence.

Scale/Domain	Primary Thermodynamic Perturbation	Dominant Mechanistic Source of Entropy (σ)	Biophysical Manifestation/Observable Signature	Quantitative Metric or Parameter	Thermodynamic Descriptor/Analytical Construct	References
Quantum–Electronic (10^−15^–10^−12^ s)	Collapse of redox coherence in electron transport	ROS/RNS-induced Fe–S cluster distortion; stochastic tunneling interruptions	Sub-femtosecond decoherence; nanoscopic heat quanta release	Coherence time τ_c ↓ 300 → 40 fs; NADH lifetime variance ↑ 4–6×	Δσₑ = k_B ln(Ω_1_/Ω_0_); Q_ℏ (coherence entropy flux)	[48,49,50]
Molecular–Mitochondrial (10^−9^–10^−3^ s)	Fractal thermal turbulence in inner membrane	Cardiolipin oxidation; proton-leak microbursts; DRP1-driven fission	Non-ergodic “thermal grains”; intermittent Δψ_m collapse	ΔT_micro ≈ 0.2–0.4 mK; Δψ_m oscillation 0.1–1 Hz	σₘᵢₜ = ∑ J_q · ∇(1/T); D_f (fractal heat dimension ≈ 2.4)	[51,52]
Sub-cellular–Cytoskeletal (10^−6^–10^−2^ s)	Piezo-thermomechanical conversion of intracellular noise	Actin filament deformation; microtubule–ion coupling; Ca^2+^ piezo-resonance	Nanometer membrane oscillations; viscoelastic noise amplification	Oscillation amplitude ↑ 250%; spectral broadening 0.1–60 Hz	σ_a_ = ∫ ζ (∂x/∂t)^2^ dt; η_eff (viscoelastic dissipation)	[53]
Cellular–Glial (ms–s)	Loss of astrocytic Ca^2+^ coherence and gap-junction symmetry	Cytokine-driven gliosis; connexin-43 fragmentation; ROS modulation	Breakdown of self-similar Ca^2+^ waves; formation of “thermal islands”	Coherence length ξ_c ↓ 300 → 60 µm; 1/f → exponential spectrum	H = ½ log (P_entropy); σ_glia (1/f entropy-power index)	[54]
Perivascular–Vascular (s–min)	Resonant vasomotor instability and thermofluidic friction	NO/ET-1 phase collapse; AQP4 mislocalization; viscosity gain	Microthermal standing waves; slowed convective clearance	Perivascular viscosity ↑ 55%; ΔT_PVS +0.3 °C	σ(g_v_) = ρ c_p ⟨(∇T)^2^⟩; Re_eff < 0.05 (laminar→chaotic)	[55,56]
Mesoscale–Network (min–h)	Phase dispersion between metabolic and electrical oscillators	NADH–BOLD desynchronization; incoherent oxygen coupling	Reduced predictive entropy; recurrent hyperemia–hypoxia cycles	Cross-freq. coherence ↓ 65%; entropy index ↑ 2.5×	S_pred = −∑ p log p; ρ_phase (phase-dispersion ratio)	[57,58]
Macroscopic–Systemic (h–days)	Flattening of hierarchical energy coupling	Spatial decoherence of entropy flux Js; vascular thermal flattening	Transition to diffusion-dominated regime; cortical spectral flattening	Fractal dimension Dt ↓ 2.6 → 2.0; 1/f slope → 0	σ_sys = ∂S/∂t; Λ_ETF (Entropy-Transfer Function)	[59,60]

**Table 2:** Multidimensional framework for the experimental quantification of neuroenergetic resilience. Each resilience dimension—elasticity, coherence, reversibility, memory, stability, and adaptivity—represents a distinct layer of the brain’s thermodynamic integrity.

Resilience Dimension	Experimental Probe/Perturbation	Measured Recovery Behavior	Key Metric or Descriptor	Interpretive Insight	References
Energetic Elasticity (capacity to absorb disturbance)	Magnetothermal SPION pulses (1–5 µJ) and plasmonic photothermal bursts (~808 nm, <1 ms)	Single → multi-exponential relaxation of temperature, viscosity, redox potential	κ (thermal conductance), τ^R^ (recovery half-time), EEI = κ/τ^R^	Rapid, coherent decay of local gradients; decline = network stiffening	[125]
Temporal Coherence (phase alignment across domains)	Sequential optical + magnetic perturbations with cross-domain recovery tracing	Phase-lag increase between τ_redox, τ_vaso, τ_elec; loss of scaling	RAI = Var(τ_i_)/⟨τ_i_⟩^2^, Δϕ (phase dispersion)	Indicates desynchronization of metabolic, vascular, and electrical feedback loops	[126]
Reversibility Geometry (shape of return in energy space)	Local photothermal perturbations mapped by nanothermometry	Asymmetric trajectories; non-linear equilibrium drift	ΔS_rev = ∫(δQ/T), A_r = τ_+_/τ_−_	Larger A_r denotes partial loss of energetic reversibility	[127]
Energetic Memory (hysteretic fatigue)	Repetitive magnetothermal cycles at fixed duty ratios	Stepwise baseline shift (ΔT_0_ ↑) and cumulative energy loss (ΔQ_h_)	EFC = ΔQ_h_·N^−1^, ΔS/Δt (entropy rate)	Quantifies irreversible dissipation; analog of material fatigue	[128]
Predictive Stability (capacity to maintain forecastable energy flow)	Finite element thermodynamic modeling with experimental input	ERR ↓, D_s_ ↓ → diffusion-dominant flow	ERR (Energy Restitution Ratio), D_s_ (entropy diffusion coefficient), DTI = f(ERR,D_s_,RAI)	Integrates multiscale reversibility; loss = onset of chaotic dissipation	[129]
Closed-Loop Adaptivity (real-time self-regulation)	Quantum-plasmonic implants with feedback micro-perturbations	Continuous modulation of τ^R^, κ, ERR; self-corrective pulses	PRB (Predictive Resilience Boundary), ΔE/Δt control gain	Demonstrates active learning of thermodynamic resilience; early fatigue detection	[130]

## Data Availability

No new data were created or analyzed in this study. Data sharing is not applicable to this article.

## References

[1] Brezzo G., Simpson J., Ameen-Ali K.E., Berwick J., Martin C. (2020). Acute Effects of Systemic Inflammation upon the Neuro-Glial-Vascular Unit and Cerebrovascular Function. Brain Behav. Immun.-Health.

[2] Song N., Mei S., Wang X., Hu G., Lu M. (2024). Focusing on Mitochondria in the Brain: From Biology to Therapeutics. Transl. Neurodegener..

[3] Ivanov A.I., Malkov A.E., Waseem T., Mukhtarov M., Buldakova S., Gubkina O., Zilberter M., Zilberter Y. (2014). Glycolysis and Oxidative Phosphorylation in Neurons and Astrocytes during Network Activity in Hippocampal Slices. J. Cereb. Blood Flow Metab..

[4] Steffan D., Pezzini C., Esposito M., Franco-Romero A. (2025). Mitochondrial Aging in the CNS: Unravelling Implications for Neurological Health and Disease. Biomolecules.

[5] Voicu V., Toader C., Șerban M., Covache-Busuioc R.-A., Ciurea A.V. (2025). Systemic Neurodegeneration and Brain Aging: Multi-Omics Disintegration, Proteostatic Collapse, and Network Failure Across the CNS. Biomedicines.

[6] Liu X., Jiang N., Zhou W. (2023). Various Energetic Metabolism of Microglia in Response to Different Stimulations. Molecules.

[7] Terzioglu M., Veeroja K., Montonen T., Ihalainen T.O., Salminen T.S., Bénit P., Rustin P., Chang Y.-T., Nagai T., Jacobs H.T. (2023). Mitochondrial Temperature Homeostasis Resists External Metabolic Stresses. eLife.

[8] Zorova L.D., Popkov V.A., Plotnikov E.Y., Silachev D.N., Pevzner I.B., Jankauskas S.S., Babenko V.A., Zorov S.D., Balakireva A.V., Juhaszova M. (2018). Mitochondrial Membrane Potential. Anal. Biochem..

[9] Gu X., Chen W., Volkow N.D., Koretsky A.P., Du C., Pan Y. (2018). Synchronized Astrocytic Ca^2+^ Responses in Neurovascular Coupling during Somatosensory Stimulation and for the Resting State. Cell Rep..

[10] Goyal M.S., Snyder A.Z. (2021). Uncoupling in Intrinsic Brain Activity. Proc. Natl. Acad. Sci. USA.

[11] Yong C., Yu Y., Wei Y., Huang G., Shu L., Gao K., Zhou E. (2025). To Reveal Biomarkers Related to Macrophage and Lactic Acid Metabolism in Renal Fibrosis and Explore Their Mechanisms. Front. Immunol..

[12] Wang X., Song X., Feng W., Chang M., Yang J., Chen Y. (2025). Advanced Nanomedicines for Treating Refractory Inflammation-Related Diseases. Nano-Micro Lett..

[13] Park B., Oh D., Kim J., Kim C. (2023). Functional Photoacoustic Imaging: From Nano- and Micro- to Macro-Scale. Nano Converg..

[14] Aristov V.V., Buchelnikov A.S., Nechipurenko Y.D. (2022). The Use of the Statistical Entropy in Some New Approaches for the Description of Biosystems. Entropy.

[15] Hubbard G., Musto A. (2025). Neuroinflammation Modulates Pathologic High Frequency Events (S36.003). Neurology.

[16] Park J.H. (2025). Neuroprotective Bioactive Compounds from Marine Algae and Their By-Products Against Cerebral Ischemia–Reperfusion Injury: A Comprehensive Review. Appl. Sci..

[17] Ghitani N., von Buchholtz L.J., MacDonald D.I., Falgairolle M., Nguyen M.Q., Licholai J.A., Ryba N.J.P., Chesler A.T. (2025). A Distributed Coding Logic for Thermosensation and Inflammatory Pain. Nature.

[18] Poljšak B., Milisav I. (2024). Decreasing Intracellular Entropy by Increasing Mitochondrial Efficiency and Reducing ROS Formation—The Effect on the Ageing Process and Age-Related Damage. Int. J. Mol. Sci..

[19] Berthiaume J.M., Kurdys J.G., Muntean D.M., Rosca M.G. (2019). Mitochondrial NAD+/NADH Redox State and Diabetic Cardiomyopathy. Antioxid. Redox Signal..

[20] Gest A.M.M., Sahan A.Z., Zhong Y., Lin W., Mehta S., Zhang J. (2024). Molecular Spies in Action: Genetically Encoded Fluorescent Biosensors Light up Cellular Signals. Chem. Rev..

[21] Lucia U., Grisolia G. (2020). Thermal Resonance and Cell Behavior. Entropy.

[22] Zivieri R., Pacini N. (2018). Entropy Density Acceleration and Minimum Dissipation Principle: Correlation with Heat and Matter Transfer in Glucose Catabolism. Entropy.

[23] Porras-Gómez M., Shoaib T., Steer D., Espinosa-Marzal R.M., Leal C. (2022). Pathological Cardiolipin-Promoted Membrane Hemifusion Stiffens Pulmonary Surfactant Membranes. Biophys. J..

[24] Boyd K.J., Alder N.N., May E.R. (2017). Buckling Under Pressure: Curvature-Based Lipid Segregation and Stability Modulation in Cardiolipin-Containing Bilayers. Langmuir ACS J. Surf. Colloids.

[25] Vurro V., Moschetta M., Bondelli G., Sardar S., Magni A., Sesti V., Paternò G.M., Bertarelli C., D’Andrea C., Lanzani G. (2023). Membrane Order Effect on the Photoresponse of an Organic Transducer. Membranes.

[26] Voorsluijs V., Avanzini F., Falasco G., Esposito M., Skupin A. (2024). Calcium Oscillations Optimize the Energetic Efficiency of Mitochondrial Metabolism. iScience.

[27] Kang Y., Kang B. (2025). Drp1-Mediated Mitochondrial Fission Exacerbates Inflammatory Responses in Intestinal Epithelial Cells: A Potential Therapeutic Target for IBD. Biochem. Biophys. Res. Commun..

[28] Chiu-Lam A., Rinaldi C. (2016). Nanoscale Thermal Phenomena in the Vicinity of Magnetic Nanoparticles in Alternating Magnetic Fields. Adv. Funct. Mater..

[29] Rossnagel K., Bauer M. (2024). Toward Direct Exploration of the Few-Femtosecond Dynamics of Electronic Coherence and Correlation in Quantum Materials Using Time- and Angle-Resolved Photoemission Spectroscopy. Crystals.

[30] Schiera G., Di Liegro C.M., Schirò G., Sorbello G., Di Liegro I. (2024). Involvement of Astrocytes in the Formation, Maintenance, and Function of the Blood–Brain Barrier. Cells.

[31] Cohen-Salmon M., Guille N., Boulay A.-C. (2025). Development of Perivascular Astrocyte Processes. Front. Neurosci..

[32] Shuman T., Aharoni D., Cai D.J., Lee C.R., Chavlis S., Page-Harley L., Vetere L.M., Feng Y., Yang C.Y., Mollinedo-Gajate I. (2020). Breakdown of Spatial Coding and Interneuron Synchronization in Epileptic Mice. Nat. Neurosci..

[33] Nolte D.D. (2024). Coherent Light Scattering from Cellular Dynamics in Living Tissues. Rep. Prog. Phys..

[34] Arango-Restrepo A., Torrenegra-Rico J.D., Rubi J.M. (2025). Entropy Production in a System of Janus Particles. Entropy.

[35] Rosic B., Dukefoss D.B., Åbjørsbråten K.S., Tang W., Jensen V., Ottersen O.P., Enger R., Nagelhus E.A. (2019). Aquaporin-4-independent Volume Dynamics of Astroglial Endfeet during Cortical Spreading Depression. Glia.

[36] Han D., Chen R., Kan H., Xu Y. (2023). The Bio-Distribution, Clearance Pathways, and Toxicity Mechanisms of Ambient Ultrafine Particles. Eco-Environ. Health.

[37] Ruiz A. (2025). Dynamic Balance: A Thermodynamic Principle for the Emergence of the Golden Ratio in Open Non-Equilibrium Steady States. Entropy.

[38] Kuśmierz Ł., Dybiec B., Gudowska-Nowak E. (2018). Thermodynamics of Superdiffusion Generated by Lévy–Wiener Fluctuating Forces. Entropy.

[39] Korpyś M., Rotkegel A., Gancarczyk A., Iwaniszyn M., Sindera K., Suwak M., Kołodziej A. (2025). Thermodynamic Irreversibility of the Flow and Transfer Phenomena Within Streamlined Structures of the Catalytic Reactors. Entropy.

[40] Venegas-Aravena P., Cordaro E.G. (2024). The Multiscale Principle in Nature (Principium Luxuriæ): Linking Multiscale Thermodynamics to Living and Non-Living Complex Systems. Fractal Fract..

[41] Lei M., Zhang T., Lu X., Zhao X., Wang H., Long J., Lu Z. (2024). Membrane-Mediated Modulation of Mitochondrial Physiology by Terahertz Waves. Biomed. Opt. Express.

[42] Wang H., Chen C., Lin Y., Tian Z., Yan Z., Zeng X., Yang Y., Lin M., Ai Q., Liu X. (2025). Connexin43 and Its Regulation of Astrocyte Gap Junction Function: Influencing Depression Progression by Mediating Electrical and Chemical Signals. CNS Neurosci. Ther..

[43] Gutiérrez M., Zhang Y., Tan J.-C. (2022). Confinement of Luminescent Guests in Metal–Organic Frameworks: Understanding Pathways from Synthesis and Multimodal Characterization to Potential Applications of LG@MOF Systems. Chem. Rev..

[44] Cruces-Solis H., Babaev O., Ali H., Piletti Chatain C., Mykytiuk V., Balekoglu N., Wenger S., Krueger-Burg D. (2021). Altered Theta and Beta Oscillatory Synchrony in a Genetic Mouse Model of Pathological Anxiety. FASEB J. Off. Publ. Fed. Am. Soc. Exp. Biol..

[45] Pereiro-Morejón J.A., Fernandez-de-Cossio-Diaz J., Mulet R. (2022). Inference of Metabolic Fluxes in Nutrient-Limited Continuous Cultures: A Maximum Entropy Approach with the Minimum Information. iScience.

[46] Sabater B. (2022). Entropy Perspectives of Molecular and Evolutionary Biology. Int. J. Mol. Sci..

[47] Deco G., Sanz Perl Y., Kringelbach M.L. (2025). Complex Harmonics Reveal Low-Dimensional Manifolds of Critical Brain Dynamics. Phys. Rev. E.

[48] Kleeorin Y., Thierschmann H., Buhmann H., Georges A., Molenkamp L.W., Meir Y. (2019). How to Measure the Entropy of a Mesoscopic System via Thermoelectric Transport. Nat. Commun..

[49] Yao Y., Lu W.L., Xu B., Li C.B., Lin C.P., Waxman D., Feng J.F. (2013). The Increase of the Functional Entropy of the Human Brain with Age. Sci. Rep..

[50] Garg M., Martin-Jimenez A., Pisarra M., Luo Y., Martín F., Kern K. (2022). Real-Space Subfemtosecond Imaging of Quantum Electronic Coherences in Molecules. Nat. Photonics.

[51] Donnarumma E., Kohlhaas M., Vimont E., Kornobis E., Chaze T., Gianetto Q.G., Matondo M., Moya-Nilges M., Maack C., Wai T. (2022). Mitochondrial Fission Process 1 Controls Inner Membrane Integrity and Protects against Heart Failure. Nat. Commun..

[52] Wen F., Gao J., Zhang G., Guo S., Zhang X., Han S., Feng X., Chen X., Hu J. (2025). ROS-DRP1-Mediated Excessive Mitochondrial Fission and Autophagic Flux Inhibition Contribute to Heat Stress-Induced Apoptosis in Goat Sertoli Cells. J. Anim. Sci. Biotechnol..

[53] Walker M., Rizzuto P., Godin M., Pelling A.E. (2020). Structural and Mechanical Remodeling of the Cytoskeleton Maintains Tensional Homeostasis in 3D Microtissues under Acute Dynamic Stretch. Sci. Rep..

[54] Mishchenko T.A., Yarkov R.S., Saviuk M.O., Krivonosov M.I., Perenkov A.D., Gudkov S.V., Vedunova M.V. (2022). Unravelling Contributions of Astrocytic Connexin 43 to the Functional Activity of Brain Neuron–Glial Networks under Hypoxic State In Vitro. Membranes.

[55] Banitalebi S., Skauli N., Geiseler S., Ottersen O.P., Amiry-Moghaddam M. (2022). Disassembly and Mislocalization of AQP4 in Incipient Scar Formation after Experimental Stroke. Int. J. Mol. Sci..

[56] Simon M., Wang M.X., Ismail O., Braun M., Schindler A.G., Reemmer J., Wang Z., Haveliwala M.A., O’Boyle R.P., Han W.Y. (2022). Loss of Perivascular Aquaporin-4 Localization Impairs Glymphatic Exchange and Promotes Amyloid β Plaque Formation in Mice. Alzheimer’s Res. Ther..

[57] Wang X., Padawer-Curry J.A., Bice A.R., Kim B., Rosenthal Z.P., Lee J.-M., Goyal M.S., Macauley S.L., Bauer A.Q. (2024). Spatiotemporal Relationships between Neuronal, Metabolic, and Hemodynamic Signals in the Awake and Anesthetized Mouse Brain. Cell Rep..

[58] Shaw K., Bell L., Boyd K., Grijseels D.M., Clarke D., Bonnar O., Crombag H.S., Hall C.N. (2021). Neurovascular Coupling and Oxygenation Are Decreased in Hippocampus Compared to Neocortex Because of Microvascular Differences. Nat. Commun..

[59] Duan J., Wei K., Du Q., Ma L., Yu H., Qi H., Tan Y., Zhong G., Li H. (2024). High-Entropy Superparaelectrics with Locally Diverse Ferroic Distortion for High-Capacitive Energy Storage. Nat. Commun..

[60] Guo J., Yu H., Ren Y., Qi H., Yang X., Deng Y., Zhang S.-T., Chen J. (2023). Multi-Symmetry High-Entropy Relaxor Ferroelectric with Giant Capacitive Energy Storage. Nano Energy.

[61] Martyushev L.M. (2025). The Significance of the Entropic Measure of Time in Natural Sciences. Entropy.

[62] Déli É., Peters J.F., Kisvárday Z. (2022). How the Brain Becomes the Mind: Can Thermodynamics Explain the Emergence and Nature of Emotions?. Entropy.

[63] Aimet S., Tajik M., Tournaire G., Schüttelkopf P., Sabino J., Sotiriadis S., Guarnieri G., Schmiedmayer J., Eisert J. (2025). Experimentally Probing Landauer’s Principle in the Quantum Many-Body Regime. Nat. Phys..

[64] Wang L., Gunduz M.A., Semeano A.T., Yılmaz E.C., Alanazi F.A.H., Imir O.B., Yener U., Arbelaez C.A., Usuga E., Teng Y.D. (2022). Coexistence of Chronic Hyperalgesia and Multilevel Neuroinflammatory Responses after Experimental SCI: A Systematic Approach to Profiling Neuropathic Pain. J. Neuroinflamm..

[65] Bauer L.G., Hirsch F., Jones C., Hollander M., Grohs P., Anand A., Plant C., Wohlschläger A. (2022). Quantification of Kuramoto Coupling Between Intrinsic Brain Networks Applied to fMRI Data in Major Depressive Disorder. Front. Comput. Neurosci..

[66] Muehsam D., Ventura C. (2014). Life Rhythm as a Symphony of Oscillatory Patterns: Electromagnetic Energy and Sound Vibration Modulates Gene Expression for Biological Signaling and Healing. Glob. Adv. Health Med..

[67] Zhang J., Ma L., Hou Y., Ouyang H., Hong H., Kim K., Kang H., Chu Z. (2025). Nanodiamond-Based Sensing: A Revolution for Biosensors in Capturing Elusive Bio-Signals in Living Cells. Adv. Drug Deliv. Rev..

[68] Lee G., Park Y.I. (2018). Lanthanide-Doped Upconversion Nanocarriers for Drug and Gene Delivery. Nanomaterials.

[69] Steinegger A., Wolfbeis O.S., Borisov S.M. (2020). Optical Sensing and Imaging of pH Values: Spectroscopies, Materials, and Applications. Chem. Rev..

[70] Yu S., Jang D., Yuan H., Huang W.-T., Kim M., Marques Mota F., Liu R.-S., Lee H., Kim S., Kim D.H. (2021). Plasmon-Triggered Upconversion Emissions and Hot Carrier Injection for Combinatorial Photothermal and Photodynamic Cancer Therapy. ACS Appl. Mater. Interfaces.

[71] Zhang F., Oiticica P.R.A., Abad-Arredondo J., Arai M.S., Oliveira O.N., Jaque D., Fernandez Dominguez A.I., de Camargo A.S.S., Haro-González P. (2024). Brownian Motion Governs the Plasmonic Enhancement of Colloidal Upconverting Nanoparticles. Nano Lett..

[72] Liao J., Silvoni S., Desch S., Serian A., Andoh J., Flor H. (2025). Cortical Activity during Painful and Non-Painful Stimulation over Four Lower Limb Body Sites: A Functional near-Infrared Spectroscopy Study. Sci. Rep..

[73] Padova D., Faria A., Ratnanather J.T., So R.J., Zhu S., Agrawal Y. (2024). Vestibular Function Is Associated with Prefrontal and Sensorimotor Cortical Gray Matter Volumes in a Cross-Sectional Study of Healthy, Older Adults. Aperture Neuro.

[74] Schirhagl R., Chang K., Loretz M., Degen C.L. (2014). Nitrogen-Vacancy Centers in Diamond: Nanoscale Sensors for Physics and Biology. Annu. Rev. Phys. Chem..

[75] Zhu Y., Cai M., Zhang Q., Wang P., Yang Y., Zhao J., Zhu W., Wang G. (2025). Quantum Sensing of Local Magnetic Phase Transitions and Fluctuations near the Curie Temperature in Tm3Fe5O12 Using NV Centers. Micromachines.

[76] Szawioła G., Mieloch S., Stefańska D., Głowacki P., Frajtak A., Michalczyk J., Murawski K., Pruchlat A., Raczyński J., Schmidt M. (2025). Thermal Effects in Optically Detected Magnetic Resonance of Nitrogen Vacancies in Diamond: A Quantum Thermometer for a Graduate Teaching Lab. J. Chem. Educ..

[77] Xiang B., Xiong W. (2024). Molecular Polaritons for Chemistry, Photonics and Quantum Technologies. Chem. Rev..

[78] Zhang Z., Jin Y., Tang J., Liu J. (2024). Simultaneously Detecting the Power and Temperature of a Microwave Sensor via the Quantum Technique. Micromachines.

[79] Zhang T., Pramanik G., Zhang K., Gulka M., Wang L., Jing J., Xu F., Li Z., Wei Q., Cigler P. (2021). Toward Quantitative Bio-Sensing with Nitrogen–Vacancy Center in Diamond. ACS Sens..

[80] Gust A., Zander A., Gietl A., Holzmeister P., Schulz S., Lalkens B., Tinnefeld P., Grohmann D. (2014). A Starting Point for Fluorescence-Based Single-Molecule Measurements in Biomolecular Research. Molecules.

[81] Adeniba O.O., Corbin E.A., Ganguli A., Kim Y., Bashir R. (2020). Simultaneous Time-Varying Viscosity, Elasticity, and Mass Measurements of Single Adherent Cancer Cells across Cell Cycle. Sci. Rep..

[82] Hülsemann M.J., Naumann E., Rasch B. (2019). Quantification of Phase-Amplitude Coupling in Neuronal Oscillations: Comparison of Phase-Locking Value, Mean Vector Length, Modulation Index, and Generalized-Linear-Modeling-Cross-Frequency-Coupling. Front. Neurosci..

[83] Yang D., Liu X., Ma J., Cui B., Wang Y., Xu J., Zhang Y., Ding H., Wang D., Liu Q. (2025). Probing Single-Cell Adhesion Kinetics and Nanomechanical Force with Surface Plasmon Resonance Imaging. ACS Nano.

[84] Veselá P., Hlubučková M., Kanclíř V., Václavík J., Žídek K. (2025). The Effect of Aging in Thin Films in the Picosecond Sonar Experiment. Sci. Rep..

[85] Richter M., Güttler A., Pauli J., Vogel K., Homann C., Würth C., Resch-Genger U. (2025). Extending Certified Spectral Fluorescence Standards for the Calibration and Performance Validation of Fluorescence Instruments to the NIR—Closing the Gap from 750 to 940 Nm with Two Novel NIR Dyes. Anal. Bioanal. Chem..

[86] Latychevskaia T., Mancini G.F., Carbone F. (2015). The Role of the Coherence in the Cross-Correlation Analysis of Diffraction Patterns from Two-Dimensional Dense Mono-Disperse Systems. Sci. Rep..

[87] Wang Z., Zhou Z., Wu M., Zhu Z. (2024). A Thermodynamic Based Constitutive Model Considering the Mutual Influence of Multiple Physical Fields. Sci. Rep..

[88] Zou H., Zhao H., Lu M., Wang J., Deng Z., Wang J. (2025). Predicting Thermodynamic Stability of Inorganic Compounds Using Ensemble Machine Learning Based on Electron Configuration. Nat. Commun..

[89] Alkahtani M., Alzahrani Y.A., Alromaeh A., Hemmer P. (2024). Novel Nanocomposites for Luminescent Thermometry with Two Different Modalities. Molecules.

[90] Revete A., Aparicio A., Cisterna B.A., Revete J., Luis L., Ibarra E., Segura González E.A., Molino J., Reginensi D. (2022). Advancements in the Use of Hydrogels for Regenerative Medicine: Properties and Biomedical Applications. Int. J. Biomater..

[91] Bock M., Reichenbach J.R. (2025). Quantum Physics at a Historic Milestone: How Has It Shaped Medical Physics?. Z. Med. Phys..

[92] Wu Y., Li F., Wu Y., Wang H., Gu L., Zhang J., Qi Y., Meng L., Kong N., Chai Y. (2024). Lanthanide Luminescence Nanothermometer with Working Wavelength beyond 1500 Nm for Cerebrovascular Temperature Imaging in Vivo. Nat. Commun..

[93] Jabbari M.B., Karamati M.R. (2022). The Effects of Temperature on the Dynamics of the Biological Neural Network. J. Biol. Phys..

[94] Zheng Q., Xie W., Lückemeyer D.D., Lay M., Wang X.-W., Dong X., Limjunyawong N., Ye Y., Zhou F.-Q., Strong J.A. (2022). Synchronized Cluster Firing, a Distinct Form of Sensory Neuron Activation, Drives Spontaneous Pain. Neuron.

[95] Dienel G.A., Rothman D.L., Mangia S. (2025). A Bird’s-Eye View of Glycolytic Upregulation in Activated Brain: The Major Fate of Lactate Is Release From Activated Tissue, Not Shuttling to Nearby Neurons. J. Neurochem..

[96] Tsukamoto M., Ito S., Ogawa K., Ashida Y., Sasaki K., Kobayashi K. (2022). Accurate Magnetic Field Imaging Using Nanodiamond Quantum Sensors Enhanced by Machine Learning. Sci. Rep..

[97] Sung D., Risk B.B., Kottke P.A., Allen J.W., Nahab F., Fedorov A.G., Fleischer C.C. (2022). Comparisons of Healthy Human Brain Temperature Predicted from Biophysical Modeling and Measured with Whole Brain MR Thermometry. Sci. Rep..

[98] Villareal J.A.B., Bathe T., Hery G.P., Phillips J.L., Tsering W., Prokop S. (2024). Deterioration of Neuroimmune Homeostasis in Alzheimer’s Disease Patients Who Survive a COVID-19 Infection. J. Neuroinflamm..

[99] Coomer C.A., Carlon-Andres I., Iliopoulou M., Dustin M.L., Compeer E.B., Compton A.A., Padilla-Parra S. (2020). Single-Cell Glycolytic Activity Regulates Membrane Tension and HIV-1 Fusion. PLoS Pathog..

[100] Alipour M., Abdolmaleki M., Shabanpour Y., Zali A., Ashrafi F., Nohesara S., Hajipour-Verdom B. (2025). Advances in Magnetic Field Approaches for Non-Invasive Targeting Neuromodulation. Front. Hum. Neurosci..

[101] Naveed M., Kim M.-H. (2025). Nanoparticle-Mediated Magnetic Hyperthermia in the Treatment of Neurological Disorders. Nanomed..

[102] Vangijzegem T., Lecomte V., Ternad I., Van Leuven L., Muller R.N., Stanicki D., Laurent S. (2023). Superparamagnetic Iron Oxide Nanoparticles (SPION): From Fundamentals to State-of-the-Art Innovative Applications for Cancer Therapy. Pharmaceutics.

[103] Attaluri A., Kandala S.K., Zhou H., Wabler M., DeWeese T.L., Ivkov R. (2020). Magnetic Nanoparticle Hyperthermia for Treating Locally Advanced Unresectable and Borderline Resectable Pancreatic Cancers: The Role of Tumor Size and Eddy-Current Heating. Int. J. Hyperth. Off. J. Eur. Soc. Hyperthermic Oncol. N. Am. Hyperth. Group.

[104] Feng W., Hussin T.A.R., Yang X. (2025). A Temperature-Indexed Concrete Damage Plasticity Framework for Thermomechanical Analysis of Concrete Structures. J. Eng. Appl. Sci..

[105] Finkelstein-Shapiro D., Mante P.-A., Sarisozen S., Wittenbecher L., Minda I., Balci S., Pullerits T., Zigmantas D. (2021). Understanding Radiative Transitions and Relaxation Pathways in Plexcitons. Chem.

[106] Pliouta L., Lampsas S., Kountouri A., Korakas E., Thymis J., Kassi E., Oikonomou E., Ikonomidis I., Lambadiari V. (2025). Mitochondrial Dysfunction in the Development and Progression of Cardiometabolic Diseases: A Narrative Review. J. Clin. Med..

[107] Jiracheewanun S., Cortie M.B., Pissuwan D. (2023). Thermal Effect during Laser-Induced Plasmonic Heating of Polyelectrolyte-Coated Gold Nanorods in Well Plates. Nanomaterials.

[108] Schawe J.E.K. (2025). Correction of Thermal Inertia on Scanning Calorimetry. Thermochim. Acta.

[109] Tsubouchi M., Hoshina H., Nagai M., Isoyama G. (2020). Plane Photoacoustic Wave Generation in Liquid Water Using Irradiation of Terahertz Pulses. Sci. Rep..

[110] Cui X., Ruan Q., Zhuo X., Xia X., Hu J., Fu R., Li Y., Wang J., Xu H. (2023). Photothermal Nanomaterials: A Powerful Light-to-Heat Converter. Chem. Rev..

[111] Bonvento G., Bolaños J.P. (2021). Astrocyte-Neuron Metabolic Cooperation Shapes Brain Activity. Cell Metab..

[112] Burmistrov I., Khanna R., Gorshkov N., Kiselev N., Artyukhov D., Boychenko E., Yudin A., Konyukhov Y., Kravchenko M., Gorokhovsky A. (2022). Advances in Thermo-Electrochemical (TEC) Cell Performances for Harvesting Low-Grade Heat Energy: A Review. Sustainability.

[113] Ndrepepa G., Kastrati A. (2023). Coronary No-Reflow after Primary Percutaneous Coronary Intervention—Current Knowledge on Pathophysiology, Diagnosis, Clinical Impact and Therapy. J. Clin. Med..

[114] Tashiro E., Kozono N., Higaki H., Shimoto T., Nakashima Y. (2024). In Vivo Shoulder Kinematic Changes and Rotator Cuff Healing after Surgical Repair of Large-to-Massive Rotator Cuff Tears. J. Orthop. Surg..

[115] Manu D.R., Slevin M., Barcutean L., Forro T., Boghitoiu T., Balasa R. (2023). Astrocyte Involvement in Blood–Brain Barrier Function: A Critical Update Highlighting Novel, Complex, Neurovascular Interactions. Int. J. Mol. Sci..

[116] Costa F.P., Wiedenmann B., Schöll E., Tuszynski J. (2024). Emerging Cancer Therapies: Targeting Physiological Networks and Cellular Bioelectrical Differences with Non-Thermal Systemic Electromagnetic Fields in the Human Body—A Comprehensive Review. Front. Netw. Physiol..

[117] Bertsch P., Diba M., Mooney D.J., Leeuwenburgh S.C.G. (2023). Self-Healing Injectable Hydrogels for Tissue Regeneration. Chem. Rev..

[118] Nesci S., Trombetti F., Pagliarani A., Ventrella V., Algieri C., Tioli G., Lenaz G. (2021). Molecular and Supramolecular Structure of the Mitochondrial Oxidative Phosphorylation System: Implications for Pathology. Life.

[119] Ruta S., Hovorka O., Huang P.-W., Wang K., Ju G., Chantrell R. (2017). First Order Reversal Curves and Intrinsic Parameter Determination for Magnetic Materials; Limitations of Hysteron-Based Approaches in Correlated Systems. Sci. Rep..

[120] Potokar M., Morita M., Wiche G., Jorgačevski J. (2020). The Diversity of Intermediate Filaments in Astrocytes. Cells.

[121] Wang X., Chen H., Xuan F. (2023). Neural Network-Assisted Probabilistic Creep-Fatigue Assessment of Hydrogenation Reactor with Physics-Based Surrogate Model. Int. J. Press. Vessels Pip..

[122] Flandoli F., Pappalettera U. (2020). Stochastic Modelling of Small-Scale Perturbation. Water.

[123] Yoshida E., Kondo M., Nakae K., Ako R., Terada S., Hatano N., Liu L., Kobayashi K., Ishii S., Matsuzaki M. (2025). Whether or Not to Act Is Determined by Distinct Signals from Motor Thalamus and Orbitofrontal Cortex to Secondary Motor Cortex. Nat. Commun..

[124] Wu J., Song S., Tian X., Wang Y., Qi X. (2023). Machine Learning-Based Prediction and Interpretation of Decomposition Temperatures of Energetic Materials. Energ. Mater. Front..

[125] Jawad H.J., Yadem A.C., Menyaev Y.A., Sarimollaoglu M., Armstrong J.N., Watanabe F., Biris A.S., Stumhofer J.S., Nedosekin D., Suen J.Y. (2022). Towards Rainbow Portable Cytophone with Laser Diodes for Global Disease Diagnostics. Sci. Rep..

[126] Schreglmann S.R., Wang D., Peach R.L., Li J., Zhang X., Latorre A., Rhodes E., Panella E., Cassara A.M., Boyden E.S. (2021). Non-Invasive Suppression of Essential Tremor via Phase-Locked Disruption of Its Temporal Coherence. Nat. Commun..

[127] Tang M., Tang K., Zhang Y., Qiu J., Chen X. (2024). Motion/Force Coordinated Trajectory Tracking Control of Nonholonomic Wheeled Mobile Robot via LMPC-AISMC Strategy. Sci. Rep..

[128] Klemenc J., Šeruga D., Nagode A., Nagode M. (2019). Comprehensive Modelling of the Hysteresis Loops and Strain–Energy Density for Low-Cycle Fatigue-Life Predictions of the AZ31 Magnesium Alloy. Materials.

[129] Ren J., Kloker M. (2022). Non-Ideal Gas Behavior Matters in Hydrodynamic Instability. Sci. Rep..

[130] Ismail M.R.M., Thangaraj M., Karmiris-Obratański P., Papazoglou E., Karkalos N. (2023). Design of Real-Time Extremum-Seeking Controller-Based Modelling for Optimizing MRR in Low Power EDM. Materials.

[131] Li P., Lee G.-H., Kim S.Y., Kwon S.Y., Kim H.-R., Park S. (2021). From Diagnosis to Treatment: Recent Advances in Patient-Friendly Biosensors and Implantable Devices. ACS Nano.

[132] Zhu Y., Tang S., Wang C., Jiang W., Zhao J., Li G. (2019). Absolute Stability Condition Derivation for Position Closed-Loop System in Hydraulic Automatic Gauge Control. Processes.

[133] Werner C., Sauer M., Geis C. (2021). Super-Resolving Microscopy in Neuroscience. Chem. Rev..

[134] García-Domínguez M. (2025). Neuroinflammation: Mechanisms, Dual Roles, and Therapeutic Strategies in Neurological Disorders. Curr. Issues Mol. Biol..

[135] Mattay R.R., Kim K., Shah L., Shah B., Sugrue L., Safoora F., Ozhinsky E., Narsinh K.H. (2024). MR Thermometry during Transcranial MR Imaging–Guided Focused Ultrasound Procedures: A Review. AJNR Am. J. Neuroradiol..

[136] Abouelregal A.E., Alharb R.A., Yaylacı M., Mohamed B.O., Megahid S.F. (2024). Analysis of Temperature Changes in Living Tissue Using the Modified Fractional Thermal Conduction Model under Laser Heat Flux on the Skin Surface. Contin. Mech. Thermodyn..

[137] Leng F., Edison P. (2021). Neuroinflammation and Microglial Activation in Alzheimer Disease: Where Do We Go from Here?. Nat. Rev. Neurol..

[138] Toader C., Dumitru A.V., Eva L., Serban M., Covache-Busuioc R.-A., Ciurea A.V. (2024). Nanoparticle Strategies for Treating CNS Disorders: A Comprehensive Review of Drug Delivery and Theranostic Applications. Int. J. Mol. Sci..

[139] Sack I. (2023). Magnetic Resonance Elastography from Fundamental Soft-Tissue Mechanics to Diagnostic Imaging. Nat. Rev. Phys..

[140] Lu Y., Kundu M., Zhong D. (2020). Effects of Nonequilibrium Fluctuations on Ultrafast Short-Range Electron Transfer Dynamics. Nat. Commun..

[141] Laquatra C., Magro A., Guarra F., Lambrughi M., Ferrone L., Fracasso G., Bacchin M., La Spina M., Moroni E., Papaleo E. (2025). Point Mutations of the Mitochondrial Chaperone TRAP1 Affect Its Functions and Pro-Neoplastic Activity. Cell Death Dis..

[142] Du S., Guan Y., Xie A., Yan Z., Gao S., Li W., Rao L., Chen X., Chen T. (2023). Extracellular Vesicles: A Rising Star for Therapeutics and Drug Delivery. J. Nanobiotechnol..

[143] Noh M.-Y., Kwon H.S., Kwon M.-S., Nahm M., Jin H.K., Bae J., Kim S.H. (2025). Biomarkers and Therapeutic Strategies Targeting Microglia in Neurodegenerative Diseases: Current Status and Future Directions. Mol. Neurodegener..

[144] Abdullah W., Razak N.N.A.N.A., Dheyab M.A., Salem F., Aziz A.A., Alanezi S.T., Oladzadabbasabadi N., Ghasemlou M. (2025). Melanin-Driven Green Synthesis and Surface Modification of Metal and Metal-Oxide Nanoparticles for Biomedical Applications. Adv. Funct. Mater..

[145] Stec-Martyna E., Wojtczak K., Nowak D., Stawski R. (2025). Battle of the Biomarkers of Systemic Inflammation. Biology.

[146] Lee S.Y., Lin M., Lee A., Park Y.I. (2017). Lanthanide-Doped Nanoparticles for Diagnostic Sensing. Nanomaterials.

[147] Vogel K., Greinert T., Reichard M., Held C., Harms H., Maskow T. (2020). Thermodynamics and Kinetics of Glycolytic Reactions. Part I: Kinetic Modeling Based on Irreversible Thermodynamics and Validation by Calorimetry. Int. J. Mol. Sci..

[148] Hernandez J.A., Ruiz A., Fonseca L.F., Pettes M.T., Jose-Yacaman M., Benitez A. (2018). Thermoelectric Properties of SnSe Nanowires with Different Diameters. Sci. Rep..

[149] Ahmed F., Muzammal Hussain M., Ullah Khan W., Xiong H. (2024). Exploring Recent Advancements and Future Prospects on Coordination Self-Assembly of the Regulated Lanthanide-Doped Luminescent Supramolecular Hydrogels. Coord. Chem. Rev..

[150] Parvin N., Joo S.W., Jung J.H., Mandal T.K. (2025). Multimodal AI in Biomedicine: Pioneering the Future of Biomaterials, Diagnostics, and Personalized Healthcare. Nanomaterials.

[151] Massaro D., Rezaeiravesh S., Schlatter P. (2023). On the Potential of Transfer Entropy in Turbulent Dynamical Systems. Sci. Rep..

[152] Martínez Lozada P.S., Pozo Neira J., Leon-Rojas J.E. (2025). From Tumor to Network: Functional Connectome Heterogeneity and Alterations in Brain Tumors—A Multimodal Neuroimaging Narrative Review. Cancers.

[153] Šimić I., Veas E., Sabol V. (2025). A Comprehensive Analysis of Perturbation Methods in Explainable AI Feature Attribution Validation for Neural Time Series Classifiers. Sci. Rep..

[154] Onciul R., Tataru C.-I., Dumitru A.V., Crivoi C., Serban M., Covache-Busuioc R.-A., Radoi M.P., Toader C. (2025). Artificial Intelligence and Neuroscience: Transformative Synergies in Brain Research and Clinical Applications. J. Clin. Med..

[155] Yu Y., Akif A., Herman P., Cao M., Rothman D.L., Carson R.E., Agarwal D., Evans A.C., Hyder F. (2023). A 3D Atlas of Functional Human Brain Energetic Connectome Based on Neuropil Distribution. Cereb. Cortex N. Y. N 1991.

[156] Joo J.I., Zhou J.X., Huang S., Cho K.-H. (2018). Determining Relative Dynamic Stability of Cell States Using Boolean Network Model. Sci. Rep..

[157] Chmiel J., Kurpas D. (2025). Burnout and the Brain—A Mechanistic Review of Magnetic Resonance Imaging (MRI) Studies. Int. J. Mol. Sci..

[158] Kölliker-Frers R., Udovin L., Otero-Losada M., Kobiec T., Herrera M.I., Palacios J., Razzitte G., Capani F. (2021). Neuroinflammation: An Integrating Overview of Reactive-Neuroimmune Cell Interactions in Health and Disease. Mediators Inflamm..

[159] Jang H., Yoon D., Nam Y. (2022). Enhancement of Thermoplasmonic Neural Modulation Using a Gold Nanorod-Immobilized Polydopamine Film. ACS Appl. Mater. Interfaces.

[160] Brandão Da Silva Assis M., Nestal De Moraes G., De Souza K.R. (2024). Cerium Oxide Nanoparticles: Chemical Properties, Biological Effects and Potential Therapeutic Opportunities. Biomed. Rep..

[161] Honciuc M., Honciuc A. (2025). Scaling Amphiphilicity with Janus Nanoparticles: A New Frontier in Nanomaterials and Interface Science. Nanomaterials.

[162] Madadi M., Khoee S. (2023). Magnetite-Based Janus Nanoparticles, Their Synthesis and Biomedical Applications. Wiley Interdiscip. Rev. Nanomed. Nanobiotechnol..

[163] Dimov I.B., Moser M., Malliaras G.G., McCulloch I. (2022). Semiconducting Polymers for Neural Applications. Chem. Rev..

[164] Mohsin M., Shams F., Li H., Alam A., Xia C., Fan L., Cao Y., Jiang W., Nasir A., Khan S. (2025). Nanozymes in Neuropathic Pain: Strategies Bridging Oxidative Stress, Mitochondrial Repair, and Neuroimmune Modulation for Targeted Therapy. J. Neuroinflamm..

[165] Martin D.R., Matyushov D.V. (2017). Electron-Transfer Chain in Respiratory Complex I. Sci. Rep..

[166] Cardoso F.d.S., Barrett D.W., Wade Z., Gomes da Silva S., Gonzalez-Lima F. (2022). Photobiomodulation of Cytochrome c Oxidase by Chronic Transcranial Laser in Young and Aged Brains. Front. Neurosci..

[167] Quirk B.J., Whelan H.T. (2020). What Lies at the Heart of Photobiomodulation: Light, Cytochrome C Oxidase, and Nitric Oxide-Review of the Evidence. Photobiomodulation Photomed. Laser Surg..

[168] Zhou H., Auerbach N., Uzan M., Zhou Y., Banu N., Zhi W., Huber M.E., Watanabe K., Taniguchi T., Myasoedov Y. (2023). Imaging Quantum Oscillations and Millitesla Pseudomagnetic Fields in Graphene. Nature.

[169] Cuadra L., Salcedo-Sanz S., Nieto-Borge J.C. (2023). Carrier Transport in Colloidal Quantum Dot Intermediate Band Solar Cell Materials Using Network Science. Int. J. Mol. Sci..

[170] Zhang Z., Dreon D., Esslinger T., Jaksch D., Buca B., Donner T. (2025). Dissipation-Induced Non-Equilibrium Phases with Temporal and Spatial Order. Commun. Phys..

[171] Freire M.A.M., Lima R.R., Bittencourt L.O., Guimaraes J.S., Falcao D., Gomes-Leal W. (2023). Astrocytosis, Inflammation, Axonal Damage and Myelin Impairment in the Internal Capsule Following Striatal Ischemic Injury. Cells.

[172] Bojarskaite L., Nafari S., Ravnanger A.K., Frey M.M., Skauli N., Åbjørsbråten K.S., Roth L.C., Amiry-Moghaddam M., Nagelhus E.A., Ottersen O.P. (2024). Role of Aquaporin-4 Polarization in Extracellular Solute Clearance. Fluids Barriers CNS.

[173] Tian J., Zhang Y., Liu L., Li C., Hao X. (2025). Deciphering Aquaporin-4′s Influence on Perivascular Diffusion Indices Using DTI in Rat Stroke Studies. Front. Neurosci..

[174] Fazio A., Neri I., Koufi F.-D., Marvi M.V., Galvani A., Evangelisti C., McCubrey J.A., Cocco L., Manzoli L., Ratti S. (2024). Signaling Role of Pericytes in Vascular Health and Tissue Homeostasis. Int. J. Mol. Sci..

[175] Mohammed D., Hamid I., Vanhollebeke B., Martin M. (2025). Microtubules and Mechanosensing: Key Players in Endothelial Responses to Mechanical Stimuli. Cell. Mol. Life Sci..

[176] Dong R., Han Y., Jiang L., Liu S., Zhang F., Peng L., Wang Z., Ma Z., Xia T., Gu X. (2022). Connexin 43 Gap Junction-Mediated Astrocytic Network Reconstruction Attenuates Isoflurane-Induced Cognitive Dysfunction in Mice. J. Neuroinflamm..

[177] Abhinav V., Basu P., Verma S.S., Verma J., Das A., Kumari S., Yadav P.R., Kumar V. (2025). Advancements in Wearable and Implantable BioMEMS Devices: Transforming Healthcare Through Technology. Micromachines.

[178] Șerban M., Toader C., Covache-Busuioc R.-A. (2025). CRISPR and Artificial Intelligence in Neuroregeneration: Closed-Loop Strategies for Precision Medicine, Spinal Cord Repair, and Adaptive Neuro-Oncology. Int. J. Mol. Sci..

[179] Sun M., Chen Y., Fan X., Li D., Song J., Yu K., Zhao Z. (2024). Electronic Asymmetry of Lattice Oxygen Sites in ZnO Promotes the Photocatalytic Oxidative Coupling of Methane. Nat. Commun..

[180] Sawada Y., Daigaku Y., Toma K. (2025). Maximum Entropy Production Principle of Thermodynamics for the Birth and Evolution of Life. Entropy.

[181] Pinardi M., Longo M.R., Formica D., Strbac M., Mehring C., Burdet E., Di Pino G. (2023). Impact of Supplementary Sensory Feedback on the Control and Embodiment in Human Movement Augmentation. Commun. Eng..

[182] Hoffmann M.H., Kirchner H., Krönke G., Riemekasten G., Bonelli M. (2024). Inflammatory Tissue Priming: Novel Insights and Therapeutic Opportunities for Inflammatory Rheumatic Diseases. Ann. Rheum. Dis..

[183] Wu R., Matta M., Paulsen B.D., Rivnay J. (2022). Operando Characterization of Organic Mixed Ionic/Electronic Conducting Materials. Chem. Rev..

[184] Ohkawara B., Ito M., Ohno K. (2021). Secreted Signaling Molecules at the Neuromuscular Junction in Physiology and Pathology. Int. J. Mol. Sci..

[185] Müller L., Di Benedetto S., Müller V. (2025). From Homeostasis to Neuroinflammation: Insights into Cellular and Molecular Interactions and Network Dynamics. Cells.

[186] Bao Y., Hu C., Wang B., Liu X., Wu Q., Xu D., Shi Z., Sun C. (2025). Mitochondrial Reverse Electron Transport: Mechanisms, Pathophysiological Roles, and Therapeutic Potential. Biology.

[187] Tang G., Yang G.-Y. (2016). Aquaporin-4: A Potential Therapeutic Target for Cerebral Edema. Int. J. Mol. Sci..

[188] Clark K.B. (2023). Neural Field Continuum Limits and the Structure–Function Partitioning of Cognitive–Emotional Brain Networks. Biology.

[189] van Merode F., Boersma H., Tournois F., Winasti W., Reis de Almeida Passos N.A., Ham A. (2025). van der Using Entropy Metrics to Analyze Information Processing Within Production Systems: The Role of Organizational Constraints. Logistics.

[190] Rué-Queralt J., Stevner A., Tagliazucchi E., Laufs H., Kringelbach M.L., Deco G., Atasoy S. (2021). Decoding Brain States on the Intrinsic Manifold of Human Brain Dynamics across Wakefulness and Sleep. Commun. Biol..

[191] Qiu H., Luo L., Su Z., Zhou L., Wang L., Chen Y. (2020). Machine Learning Approaches to Predict Peak Demand Days of Cardiovascular Admissions Considering Environmental Exposure. BMC Med. Inform. Decis. Mak..

[192] Kaushal J.B., Raut P., Kumar S. (2023). Organic Electronics in Biosensing: A Promising Frontier for Medical and Environmental Applications. Biosensors.

[193] Al Abdul Wahid S., Asad A., Mohammadi F. (2024). A Survey on Neuromorphic Architectures for Running Artificial Intelligence Algorithms. Electronics.

[194] Abid I., González-Colsa J., Naveaux C., Campu A., Arib C., Focsan M., Albella P., Edely M., Lamy de La Chapelle M. (2024). Correlation between Plasmonic and Thermal Properties of Metallic Nanoparticles. Nanomaterials.

[195] Mas-Bargues C., Huete-Acevedo J., Arnal-Forné M., Sireno L., Pérez V., Borrás C. (2025). Extracellular Vesicles as Epigenetic Regulators of Redox Homeostasis: A Systematic Review and Meta-Analysis. Antioxidants.

[196] Gao W., Yan Z., Zhou H., Xie Y., Wang H., Yang J., Yu J., Ni C., Liu P., Xie M. (2025). Revolutionizing Brain–computer Interfaces: Overcoming Biocompatibility Challenges in Implantable Neural Interfaces. J. Nanobiotechnol..

[197] Vecchio F., Miraglia F., Pappalettera C., Orticoni A., Alù F., Judica E., Cotelli M., Rossini P.M. (2021). Entropy as Measure of Brain Networks’ Complexity in Eyes Open and Closed Conditions. Symmetry.

[198] Schneider D.M., Zanette D.H. (2025). The Structure of Bit-String Similarity Networks. Entropy.

[199] Truong N.C.D., Wang X., Wanniarachchi H., Liu H. (2022). Enhancement of Frequency-Specific Hemodynamic Power and Functional Connectivity by Transcranial Photobiomodulation in Healthy Humans. Front. Neurosci..

[200] Loeb G.E. (2023). Remembrance of Things Perceived: Adding Thalamocortical Function to Artificial Neural Networks. Front. Integr. Neurosci..

[201] Rad D., Maier M., Triff Z., Marcu R. (2025). Entangled Autopoiesis: Reframing Psychotherapy and Neuroscience Through Cognitive Science and Systems Engineering. Brain Sci..

[202] Karalis V.D. (2024). The Integration of Artificial Intelligence into Clinical Practice. Appl. Biosci..

[203] Murman D.L. (2015). The Impact of Age on Cognition. Semin. Hear..

[204] Walzik D., Wences Chirino T.Y., Zimmer P., Joisten N. (2024). Molecular Insights of Exercise Therapy in Disease Prevention and Treatment. Signal Transduct. Target. Ther..

[205] Matarèse B.F.E., Rusin A., Seymour C., Mothersill C. (2023). Quantum Biology and the Potential Role of Entanglement and Tunneling in Non-Targeted Effects of Ionizing Radiation: A Review and Proposed Model. Int. J. Mol. Sci..

[206] Naser M.Z. (2025). Intuitive Tests to Validate Machine Learning Models against Physics and Domain Knowledge. Digit. Eng..

[207] Mamoudou H., Mune M.A.M. (2025). AI-Driven Bioactive Peptide Discovery of next-Generation Metabolic Biotherapeutics. Appl. Food Res..

[208] Zanin M., Aitya N.A.A., Basilio J., Baumbach J., Benis A., Behera C.K., Bucholc M., Castiglione F., Chouvarda I., Comte B. (2021). An Early Stage Researcher’s Primer on Systems Medicine Terminology. Netw. Syst. Med..

